# Global variations in critical drought thresholds that impact vegetation

**DOI:** 10.1093/nsr/nwad049

**Published:** 2023-02-24

**Authors:** Xiangyi Li, Shilong Piao, Chris Huntingford, Josep Peñuelas, Hui Yang, Hao Xu, Anping Chen, Pierre Friedlingstein, Trevor F Keenan, Stephen Sitch, Xuhui Wang, Jakob Zscheischler, Miguel D Mahecha

**Affiliations:** Sino-French Institute for Earth System Science, College of Urban and Environmental Sciences, Peking University, Beijing 100871, China; Sino-French Institute for Earth System Science, College of Urban and Environmental Sciences, Peking University, Beijing 100871, China; Key Laboratory of Alpine Ecology and Biodiversity, Institute of Tibetan Plateau Research, Center for Excellence in Tibetan Earth Science, Chinese Academy of Sciences, Beijing 100085, China; UK Centre for Ecology and Hydrology, Wallingford OX10 8BB, UK; CSIC, Global Ecology Unit CREAF-CSIC-UAB, Barcelona 08193, Spain; CREAF, Cerdanyola de Vallès, Barcelona 08193, Spain; Sino-French Institute for Earth System Science, College of Urban and Environmental Sciences, Peking University, Beijing 100871, China; Department of Biogeochemical Integration, Max Planck Institute for Biogeochemistry, Jena 07745, Germany; Sino-French Institute for Earth System Science, College of Urban and Environmental Sciences, Peking University, Beijing 100871, China; Department of Biology, Colorado State University, Fort Collins, CO 80523, USA; Graduate Degree Program in Ecology, Colorado State University, Fort Collins, CO 80523, USA; Department of Mathematics, College of Engineering, Mathematics and Physical Sciences, University of Exeter, Exeter EX4 4QF, UK; Laboratoire de Météorologie Dynamique, Institut Pierre-Simon Laplace, CNRS-ENS-UPMC-X, Département de Géosciences, Ecole Normale Supérieure, Paris 75005, France; Earth and Environmental Sciences Area, Lawrence Berkeley National Laboratory, Berkeley, CA 94720, USA; Department of Environmental Science, Policy & Management, University of California Berkeley, Berkeley, CA 94720, USA; Department of Geography, College of Life and Environmental Sciences, University of Exeter, Exeter EX4 4QF, UK; Sino-French Institute for Earth System Science, College of Urban and Environmental Sciences, Peking University, Beijing 100871, China; Department of Computational Hydrosystems, Helmholtz Centre for Environmental Research—Ufz, Leipzig 04318, Germany; Remote Sensing Centre for Earth System Research, Leipzig University, Leipzig 04103, Germany

**Keywords:** drought threshold, inflection points, vegetation response, soil moisture, drought impacts

## Abstract

Identifying the thresholds of drought that, if crossed, suppress vegetation functioning is vital for accurate quantification of how land ecosystems respond to climate variability and change. We present a globally applicable framework to identify drought thresholds for vegetation responses to different levels of known soil-moisture deficits using four remotely sensed vegetation proxies spanning 2001–2018. The thresholds identified represent critical inflection points for changing vegetation responses from highly resistant to highly vulnerable in response to drought stress, and as a warning signal for substantial vegetation impacts. Drought thresholds varied geographically, with much lower percentiles of soil-moisture anomalies in vegetated areas covered by more forests, corresponding to a comparably stronger capacity to mitigate soil water deficit stress in forested ecosystems. Generally, those lower thresholds are detected in more humid climates. State-of-the-art land models, however, overestimated thresholds of soil moisture (i.e. overestimating drought impacts), especially in more humid areas with higher forest covers and arid areas with few forest covers. Based on climate model projections, we predict that the risk of vegetation damage will increase by the end of the twenty-first century in some hotspots like East Asia, Europe, Amazon, southern Australia and eastern and southern Africa. Our data-based results will inform projections on future drought impacts on terrestrial ecosystems and provide an effective tool for drought management.

## Teaser

This study creates a highly novel data-led framework that identifies the critical inflection points for vegetation responses to increasing drought stress and develops the first global map of the drought thresholds.

## INTRODUCTION

Drought will become more intense, frequent and longer-lasting in many regions around the world with increasing global warming [1], posing risks to ecosystem health and societal welfare [2–4]. Yet, not all droughts reduce biome productivity, as vegetation resistance can mitigate negative drought impacts for some ecosystem types more so than others [5–7]. On the other hand, resistance is limited up to the highest drought stress that vegetation can tolerate. Once drought increases further and crosses a critical threshold, an amplification of vegetation response is expected. The divergent vegetation responses to drought are known to vary across biomes [7] and induce non-linear responses, e.g. due to carry-over effects [8,9], both making it challenging to assess drought impacts on global terrestrial ecosystems.

Many previous studies have evaluated the ability of an ecosystem to resist drought by linking vegetation growth to potential driving factors or detecting impacts on vegetation growth relative to normal conditions. A previous study [10] proposed a vegetation sensitivity index associated with vegetation memory effects and climate forcings based on an autoregressive modeling approach, empirically estimating the resilience of an ecosystem to global climate variability. However, while advancing the general understanding of ecological response to environmental perturbations, that study did not derive specific thresholds for drought onset. Moreover, many metrics on resistance or resilience have been developed and applied to assess vegetation response to drought, which have made much-needed investigations on the ability of divergent ecosystems to withstand drought stress and promoted the understanding of the underlying mechanisms [11–13]. Unfortunately, there is still a knowledge gap in distinguishing the climate effects and vegetation capacity by considering coupled climate–ecosystem dynamics. The aim of our study is to overcome the limitation via a percentile-based threshold that considers both the climate anomaly and vegetation response. Moreover, in contrast to some of the metrics conditioned for use in a specific drought event, we intend to provide the general applicable critical threshold for the shifts of vegetation response to drought. As such, our study is expected to reduce uncertainty in assessing drought impacts on vegetation growth in a changing climate.

**Figure 1.**
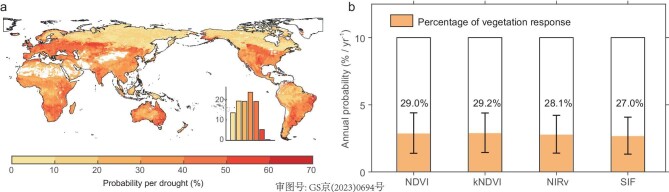
Drought-induced decreases in vegetation in years of drought occurrence during 2001–18. (a) Average probability of growing-season vegetation anomalies lower than the threshold of the 10th percentile when drought years are identified by <10th percentile of anomalies of growing-season drought indicators. Drought occurrences are identified by the moisture contents of surface soil (SMsurf). Vegetation declines are detected based on four satellite observations: normalized difference vegetation index (NDVI), kernel NDVI (kNDVI), near-infrared reflectance of vegetation (NIRv) and solar-induced chlorophyll fluorescence (SIF). The white areas in (a) represent the absence of values due to the lack of vegetation cover. (b) Overall probabilities of drought occurrence per year (open bars) and of being in both a drought year and when the vegetation has declined (colors) for kNDVI, NDVI, NIRv and SIF (27.0%–29.2% on average for occurrence of vegetation response per drought year). The probabilities are global and derived from all grids for which values are available. The error bars are indicated by the standard deviation of all probability values. The numbers on bars in (b) represent coincidence probabilities for vegetation response in each drought year.

To examine the global response of biome productivity to drought, we first investigate different vegetation responses to drought using anomalies of soil moisture from the Global Land Evaporation Amsterdam Model (GLEAM) data set and multiple remotely sensed vegetation proxies that describe vegetation ‘greenness’ and productivity (Fig. 1; ‘Methods’ section). They are the normalized difference vegetation index (NDVI) and its non-linear generalization, kernel NDVI (kNDVI) [14]; the near-infrared reflectance of vegetation (NIRv) and solar-induced chlorophyll fluorescence (SIF) [15]. Using the 10th percentile to define both drought occurrence and a pronounced vegetation suppression in response to drought in the growing season, we derive the coincidence probability for vegetation suppression per drought year (Fig. 1a). If there is a simple one-to-one mapping between levels of drought intensity and greenness suppression, then the coincidence probabilities would be near 100%. Instead, our results suggest that major drought does not necessarily translate into a noticeable vegetation response. Indeed, vegetation shows a sizeable reduction in greenness and productivity only for <30% on average of these 1-in-10 drought events during 2001–18, and even lower in regions like the Amazonia, the Congo basin and boreal areas (Fig. 1).

Hence, a globally unified quantitative definition of detrimental drought impacts on vegetation based on percentiles alone does not work. In addition, the severity of drought that suppresses vegetation varies considerably across different regions and biomes [16–18]. For instance, vegetation activity might be highly adapted to a permanent state of water scarcity leading to a low probability of reduced greenness during drought in some arid zones. For a global assessment of drought ecosystem impacts, a critically important question is therefore: What are the drought thresholds that are associated with a high coincidence of vegetation growth suppression potentially leading to widespread vegetation mortality and how do they vary spatially?

We create an observation-based function that links vegetation response to different percentile levels of negative soil-moisture anomalies (Equations (1) and (2) and Supplementary Fig. S1). We use <10th as the threshold for defining vegetation response to drought because the 10th percentile is a threshold universally set in several studies about vegetation productivity extremes [19,20] and corresponds to a wide occurrence of vegetation suppression. As illustrated in Supplementary Fig. S2, a workflow combining principal component analysis (PCA), the coincidence analysis and the detection of inflection points using segment regression are carried out in an orderly manner in this study; all are based on experiences from earlier studies of extreme events and detecting ecological thresholds [21–23]. Feature extraction for global lands is first undertaken through PCA (Supplementary Figs S3 and S4), which is designed to overcome the difficulty in limited years of data for point-by-point detection and a single global threshold that loses geographical information [22]. By doing so, the dominant modes are determined at regional to global scales to find grid cells with comparable climate conditions and vegetation dynamics.

### The applicable framework to identify drought thresholds

To describe drought thresholds, we investigate the non-linear relationship between vegetation response and drought intensity. The main functional feature of this relationship is an inflection point, which is the threshold that marks the start of intensifying drought impacts and defines the drought stress at which level vegetation suppression starts shifting (Supplementary Figs S1, S5 and S6). We base this assumption of non-linearity on both physiological theory and emerging multiple reports of different phases for vegetation responses to increasing drought stress [24–30]. For those areas with non-linear relationships (82%–86% of global vegetated areas), we derive the response function as a fractional coincidence *r* of vegetation suppression when soil moisture is at or below different soil-moisture deficit levels (Equations (1) and (2)). According to Equation (1), the values of *r* range from 0 to 1, describing a coincidence between drought and the anomalous vegetation response from none to a complete drought response [21,22].

In our framework, a relatively stable and low coincidence rate of vegetation response to increasing drought severity characterizes a Phase A response. In this phase, we suggest that vegetation has a strong capacity to withstand slight-to-mild soil water deficits. We also have a Phase B response, which is a phase of ‘rapid response’ when the coincidence rate of drought-vegetation anomaly increases substantially and steeply as droughts intensify beyond *T_S__Msurf_* (Supplementary Fig. S1). The inflection point *T_SMsurf_* that delimits the two phases is a threshold capturing the non-linear change of vegetation response from Phase A to Phase B, as vegetation adjusts from a slow to a rapid rate of increasing impacts for rising drought. We examine the performance of the non-linear response trajectory for different biomes (Supplementary Fig. S5) and verify that the *T_SMsurf_* can be detected.

### Spatial patterns of drought thresholds

**Figure 2.**
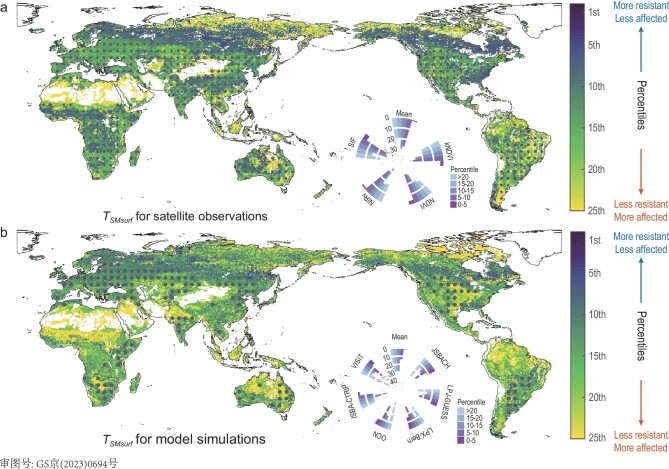
Global map of drought threshold (*T_SMsurf_*) for vegetation response to surface soil-moisture anomalies during the growing season. (a) The main map of thresholds aggregated over different combinations of four satellite-based vegetation indicators and moisture content of surface soil (SMsurf) for 2001–18. Droughts are identified by anomalies of SMsurf, and changes in vegetation activity and greenness are identified by anomalies of NDVI, kNDVI, NIRv and SIF. The areas averaged by more than one single satellite data set and model simulations with maximum coincidence rates of >0.3 are marked by dots. Lower percentiles correspond to the definition of a more severe soil water deficit as drought. Under the same degree of vegetation suppression, lower percentiles for thresholds imply locations where vegetation is more resistant to drought and so affected less by arid conditions while the higher percentiles are the opposite. The inset on the right shows the distributions of satellite thresholds as small histograms for the individual combinations of SMsurf and vegetation indicators (NDVI, kNDVI, NIRv and SIF). The numbers of 0, 10, 20 and 30 in the small histograms represent the percentages of vegetated areas in different percentile subranges (b). The same format as for (a), but the spatial patterns of aggregated threshold are from model simulations during 2001–18. Droughts are identified by the same anomalies of SMsurf as in (a), but the vegetation activity is estimated by anomalies of leaf area index (LAI) from six dynamic global vegetation models (DGVMs) in the TRENDY ensembles (JABACH, LPJ-GUESS, LPX-Bern, OCN, ISBA-CTRIP and VISIT).

**Figure 3.**
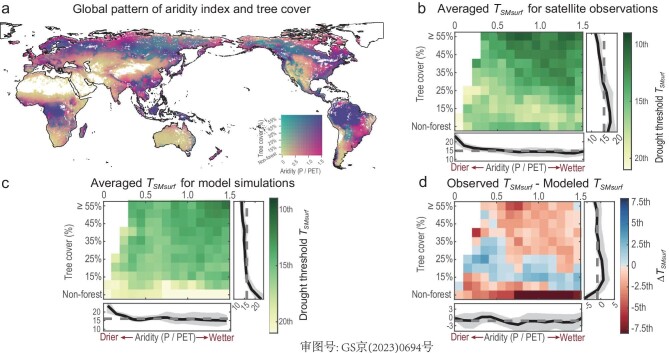
Patterns of drought threshold (*T_SMsurf_*) along the gradients of aridity and tree cover. (a) Map for different levels of aridity (horizontal axis of the color palette) and fractions of tree cover (vertical axis of the color palette). (b) Main panel: variation in averaged drought thresholds derived from satellite observations as a function of different levels of the aridity index measured by mean annual P/PET (*x*-axis) and the fraction of tree cover (*y*-axis). Non-forest is defined as <1% of tree cover. Right: change in drought thresholds along the fraction of tree cover for the means (line) and variations (shaded areas) across all levels of aridity (0–1.5). Lower: change in drought thresholds along the aridity index for the means (line) and variations (shaded areas) across all ranges of tree cover. Drier areas are denoted by smaller values of the aridity index and vice versa. Smaller percentiles of thresholds indicate that a higher drought stress level should be reached to cause a pronounced vegetation response. (c) Same format as for (b), but the variation in average drought thresholds is derived from model simulations. (d) Same format as for (b), but the difference is between satellite observations and model simulations. The positive and negative values suggest the underestimation and overestimation of drought thresholds by model simulations, respectively.

We find that *T_SMsurf_* from satellite-derived vegetation indices occur at values >10th percentile of soil water anomalies in ∼70% of areas (Fig. 2a and Supplementary Fig. S7). This suggests the shifts of vegetation response are prone to occur even when the drought stress is smaller than the severity of those 1-in-10 drought events in many locations. Our satellite-based *T_SMsurf_*, although locally consistent between nearby points, does have strongly geographical variation (Fig. 2a). For places having thresholds of lower percentile values, a transition to a vegetation response to drought is induced by higher levels of drought stress. This higher resistance corresponds to a stronger capacity for vegetation to mitigate soil water deficit and reduce sensitivity to drought. Generally, we find that observer-based *T_SMsurf_* have a more valid and stronger gradient pattern along the forest cover than the aridity, decreasing from low to high fractions of forest covers (Fig. 3a and b), demonstrating that forests are more drought-resistant. The highest percentile of drought thresholds (>15th on average) appears where there is <25% of trees, irrespective of whether in arid or humid areas. This interesting finding suggests that vegetated areas covered by little forest have limited capacity to cope with additional water stress due to the universality that the roots of other vegetation have insufficient depth to access deeper soil water [31,32]. Meanwhile, this within-year response may not capture the lag effects while it is well known that many forest ecosystems may react with substantial lag times to stress events [33,34]. By contrast, the lowest drought thresholds are for more humid regions covered by medium (>30%) to high (>50%) tree cover, such as in the Amazon, the Congo, eastern USA, southern China and part of Siberia (Figs 2a and 3a and b).

Furthermore, drought thresholds are suggested to vary in different biomes (Supplementary Fig. S8). The slight difference between *T_SMsurf_* of forests (13.0th percentile on average) and grasses (14.4th percentile on average, Supplementary Fig. S9a) is in line with the expectation that woody structures and deep forest roots [35,36] allow a better ability to cope with drought years [36–38]. Irrespective of whether located in tropical, temperate or boreal climatic regions, the *T_SMsurf_* values for forests are slightly lower (i.e. less likely to cross a stress threshold) than those of grasses, additionally supporting our finding of decreasing thresholds with increasing forest covers. Notably, for the same vegetation type, both forest and grass in tropical climates have much higher *T_SMsurf_* than in temperate and boreal climates, possibly associated with a higher sensitivity of vegetation in tropical areas to drought stress. Moreover, crop *T_SMsurf_* decreases with increasing irrigation, which reveals that agricultural management is an effective measure for alleviating the negative drought impacts. Although intuitive, we verify that higher levels of irrigation can postpone the arrival of the shift of vegetation response to drought (Supplementary Fig. S8b).

Nevertheless, the observationally based *T_SMsurf_* values have some uncertainties and especially in some tropical regions (e.g. the Amazonia) and cold areas with larger difference among different vegetation proxies (Supplementary Figs S7 and S9a). We find much smaller coincidence rates (maximum value < 0.3, Supplementary Fig. S10) in those areas than other regions. Given that drought thresholds are estimated tightly depending on the observation records, the shift of vegetation response to drought may not actually occur if the coincidence rates remain low, thereby adding to the uncertainty. According to our finding in Fig. 3, we infer that strong resistance of vegetation to drought is the main reason for the low coincidence rates, consistently with having high forest covers in these areas.

In addition to vegetation resistance, there are several possible reasons for uncertainties in tropical regions. First, some tropical regions generally feature water surplus throughout the year so low coincidence rates are detected even though those humid regions are theoretically more sensitive to drought [39]. Second, an increase in greenness during the dry season can result from the structural change of forest canopies (e.g. decreased shadow of high trees and leaf abscission). Thus, a decrease in vegetation greenness may not timely be observed under drought [40–42] and the response of greenness and photosynthesis to drought stress might decouple. Consequently, mean *T_SMsurf_* for greenness (NDVI and kNDVI) might underestimate drought thresholds when comparing *T_SMsurf_* for vegetation photosynthesis (SIF, gross primary production (GPP) and leaf area index (LAI), Supplementary Figs S7 and S11a and b). In particular, we find much higher percentiles of *T_SMsurf_* for GPP and LAI in the Congo (Supplementary Fig. S11a and b), possibly because long-term increase in dry season length reduces the photosynthesis of the Congo rainforest [43]. By contrast, *T_SMsurf_* for NDVI, kNDVI, NIRv and SIF are lower in the Congo rainforest owing to an observed increase in greenness under drought (e.g. NDVI [40]) or a response lag (e.g. SIF [44]) to drought.

To verify our main finding on the drought threshold map, we also obtain a similar spatial pattern of drought thresholds over a longer time period (1982–2018) using GIMMS NDVI_3g_ data (Supplementary Fig. S11c). While the systematic biases in the satellite sensor for the pre-2000 time series [45] probably cause the difference (i.e. lower magnitudes of thresholds in >60% of grid cells for GIMMS NDVI_3g_) compared to the entire growing-season *T_SMsurf_* (Fig. 2a), the averaged *T_SMsurf_* for each month slot within the growing season has higher magnitudes in ∼54% of grid cells (Supplementary Fig. S12). We thus infer that vegetation response to drought might vary at different timescales since drought durations can affect the drought intensities of the whole growing season. Besides, the mean response of the vegetation during the growing season could be partly offset by changing month-to-month vegetation responses to the same levels of drought. In the other direction, areas like the Amazon and the Congo show higher growing-season *T_SMsurf_* than the averaged monthly *T_SMsurf_*, which instead suggests a stronger capacity for humid rainforests to buffer against short-term drought stress.

### Models underestimate drought thresholds

*T_SMsurf_* values estimated by an ensemble of global dynamic vegetation models (DGVMs) (Supplementary Table S1) show large uncertainty among different models (Supplementary Fig. S9b). By comparison, modeled *T_SMsurf_* is suggested to overestimate satellite-derived *T_SMsurf_* in ∼58% of vegetated areas (Fig. 2a versus b), mainly in semi-humid to humid areas with medium to high forest cover and in the arid areas with very little forest cover (Fig. 3). This suggests that DGVMs may overestimate drought impacts on vegetation as modeled vegetation suppression will occur even if the drought stress is much slighter compared with observations.

This finding indicates the poor characterization of drought-related physiological mechanisms in models (e.g. [46]). Modeled *T_SMsurf_* have a weaker forest cover gradient than satellite-derived *T_SMsurf_* (Fig. 3c). The spatial variations of modeled *T_SMsurf_* are thus suggested to be less driven by self-regulation from different forest covers relative to climatic water availability. In areas with high forest cover, model simulations tend to underestimate the positive effects of physiological regulations on mitigating drought damage, e.g. by setting a fixed modeled rooting depth. The relevant empirical parameters of modeled root depth may cause the calculated overestimates of vegetation sensitivity to soil water stress [47,48], whereas in arid and semi-arid areas with low forest cover, model simulations are likely to underestimate the role of the adaptation or acclimation of vegetation to regular soil water shortage, causing an easier response shift to increasing drought stress. Notably, higher modeled *T_SMsurf_* in humid regions with non-forest land covers is likely to result from very limited grid cells instead of implying a larger difference of modeled *T_SMsurf_* deviating from observations (Fig. 3c and d). Meanwhile, DGVMs are known to underestimate the sensitivity of vegetation transpiration to high vapour pressure deficit (VPD) levels in warm droughts, predominantly due to their lack of detailed plant hydraulic function [49]. We suggest that this is a contributing factor to why modeled *T_SMsurf_* is frequently too low (i.e. having less drought damage) in some semi-arid regions at mid-to-low latitudes that have less forest cover (15%–35%).

### Future risks of crossing *T_SMsurf_*

**Figure 4.**
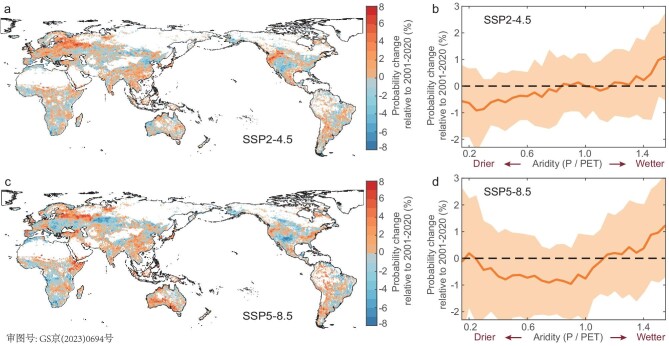
Patterns of annual probability for *T*-inferred drought occurrence in the future two decades 2081–2100 relative to the recent two decades 2001–20 under SSP2-4.5 and SSP5-8.5. (a) Spatial distribution of probability change for drought occurrence per year during 2081–2100 relative to the historical period of 2001–20 under SSP2-4.5, derived from ensemble means of 12 earth-system models (ESMs) (see Supplementary Table S2). (b) The average change in probability for drought occurrence per year during 2081–2100 along the gradients of aridity (mean annual P/PET of 2081–2100, *x*-axis) relative to the historical period of 2001–20 under SSP2-4.5. Non-forest is defined as <1% of tree cover. (c) and (d) Same format as for (a) and (b), but under SSP 5-8.5. Soil-moisture content for the 0- to 10-cm layer (CMIP6 variable name: ‘mrsos’) was used as an indicator of future drought. The variable ‘mrsos’ was interpolated to a reference spatial resolution of 0.5° × 0.5° to match the satellite-derived drought thresholds and for mapping purposes.

As we verify above, in many instances, DGVMs fail to reproduce the measurement-based values of *T_SMsurf_* but it is still worthwhile to derive the changes in soil-moisture-measured droughts from earth-system models (ESMs) based on the observe-based *T_SMsurf_*. Twelve ESMs are used from CMIP6 to quantify any probabilistic change of droughts, defined by crossing the data-based threshold *T_SMsurf_* (hereafter referred to as *T_SMsurf_*-inferred droughts) between two decades of history (2001–20) and future (2081–2100) under SSP2-4.5 and SSP5-8.5 (Fig. 4) [50].

Under SSP2-4.5, *T_SMsurf_*-inferred droughts are projected to increase in half of the vegetated areas where *T_SMsurf_* is robustly detectable in present-day measurements. We find particularly high risks of raised *T_SMsurf_*-inferred droughts in key hotspots of Europe, East Asia, eastern and northern North America and some areas of the Amazon, Australia and Africa (Fig. 4). By the end of the twenty-first century, we estimate that the largest increase in the occurrence probability of *T_SMsurf_*-inferred droughts will reach >3% in those hotspots (Fig. 4a), while *T_SMsurf_*-inferred droughts will generally increase in more humid regions, especially where there are aridity index values of >1.0, projected by >70% of ESMs in humid regions (Fig. 4b). The increases in drought frequency in more humid regions could be caused by the rising-temperature-induced intensification of evapotranspiration (ET), which exacerbates the soil water deficit, whereas ET is suppressed in more arid areas, in tandem with increased water use efficiency under higher atmospheric CO_2_.

Of particular interest is that the occurrence probability for *T_SMsurf_-*inferred droughts does not become higher in regions like Europe and western North America under the high-emission scenario of SSP5-8.5 (Fig. 4c and d), probably due to a balance between increased temperatures and precipitation levels. This finding indicates that increases in atmospheric CO_2_ concentrations may partly ameliorate the impacts of future drought stress by increasing the water use efficiency of vegetation [51]. This CO_2_ effect thus impacts the projected soil moisture and partially offsets any increases in crossing the drought thresholds of soil moisture [52]. The exceptions to this lack of change are for some hotspots like the Amazon, Australia and eastern Africa, as already noted (Fig. 4c and d). Future drought conditions are thus suggested to have a relatively smaller change when characterized by soil moisture alone [53,54].

We note that the changes in occurrence probability for future droughts are underestimated using a universal 10th percentile instead of using *T_SMsurf_*, especially marked in Europe, eastern North America and South America (Supplementary Fig. S13). Our framework for threshold detection therefore has important implications for accurately quantifying the impacts of future climates on vegetation development. The understanding of climate–ecosystem dynamics under drought is particularly much-needed for those regions that are projected to have rapid increases in extreme heat event frequency and for dryland locations that, by definition, are already subjected to drought stress. However, caveats concern the impacts of data quality, response scaling, different durations of drought within the growing season and possible acclimation and adaptation of vegetation to climate change (see more in the Supplementary discussion). Besides, this data-led method for identifying thresholds is limited by observations so the complicated non-linearity between drought stress and vegetation response needs further exploration.

In summary, our study develops a diagnostic drought threshold that describes the critical inflection point for vegetation responses to drought. Drought thresholds tend to vary geographically with the fraction of forest cover. The global variations of drought thresholds provide a unique perspective for investigating drought impacts on vegetation worldwide. Moreover, model evaluation on thresholds revealed that the state-of-the-art vegetation models tended to overestimate the negative drought impacts on ecosystem structure and functioning, such as their vegetation productivity and terrestrial carbon cycle, particularly in humid ecosystems with high forest cover. Although our results find that humid ecosystems are less affected if they are covered by more forest, future increased risks of drought in those hotspots call for more attention towards humid ecosystems, especially to those contributing a lot to global carbon sinks, human livelihood and economic development under future climate change. We recommend advancing satellite observations and improving model projections by refining biological processes in order to develop effective ecosystem policies and drought management strategies to alleviate future drought stress.

## METHODS

### Drought and aridity metrics

#### Drought indicators

The moisture contents of surface soil are obtained from the satellite-derived GLEAM data set (version v3.3a) [55]. The monthly soil-moisture data are aggregated from the original spatial resolution of 0.25° × 0.25° to the unified spatial resolution of 0.5° × 0.5° that we use throughout for all variables and averaged for the growing seasons of each year. We define the growing season using the existing data set developed by Zhu *et al.* [56] (see Supplementary Fig. S14), which is first determined from the GIMMS LAI_3g_ data set in 1981–2009 using a Savitzky–Golay filter and then refined by excluding the ground-freeze period identified by the Freeze/Thaw Earth System Data record [57]. The anomalies of growing-season drought indicators are detrended by subtracting the linear trend for 2001–18.

#### Aridity index

We calculate a simple index of background aridity to assess the projections of historical and future vegetation responses to drought. Our index is a function of the amount of background dryness or wetness (Figs 3 and 4). The aridity index is derived as the multiyear average (2001–18) of the ratio of annual precipitation (P) to annual potential evapotranspiration (PET) and using values from the CRU TS4.04 data set [58]. Values of the aridity index increase between arid and humid regions. Values of <0.65 we refer to as drylands, with 0.2–0.5 defined as semi-arid regions and 0.5–0.65 defined as semi-humid regions [59].

### Vegetation indicators

#### Satellite observations

NDVI is used as a reliable surrogate measure of vegetation greenness, broadly representing chlorophyll content and canopy structure. Monthly NDVI for 2001–18 is derived from the Moderate Resolution Imaging Spectroradiometer (MODIS) Collection 6 MOD13C2 with the native spatial resolution 0.05 × 0.05° (https://lpdaac.usgs.gov/products/mod13c2v006/). MODIS NDVI is aggregated to 0.5 × 0.5° grid resolution, averaging within each such grid, the NDVI values in all subpixels, which is also applied for other satellite data. Very low monthly values (<0.1) are removed from our analysis to exclude areas of barren, rock, sand (i.e. deserts) or snow. We also use kNDVI, simply written as kNDVI = tanh (NDVI^2^). This vegetation index is a non-linear version of NDVI, developed by a kernel methods framework. kNDVI is believed to reduce saturation effects and enhance robustness to noise [14].

Our third estimate of vegetation dynamics uses data from NIRv [60]. The NIRv version is MODIS 16-day NDVI and NIR reflectance (NDVI × NIR), derived from the MCD43C4 Vegetation Index Product with a spatial resolution of 0.05°. All values where NIRv ≤ 0 are excluded, following the threshold of Badgley *et al.* [60].

We obtain a fourth estimate of the state of the vegetation from SIF, which is a measure of the amount of light emitted by chlorophyll, itself a proxy of photosynthetic activity [61]. We use a gridded global contiguous SIF (CSIF) data set, reconstructed from SIF observations from the Orbiting Carbon Observatory-2 (OCO-2) and using a machine-learning approach [15]. This clear-sky set of instantaneous CSIF data has a 0.05° spatial and 4-day temporal resolution.

To remove the possibility that the different thresholds for satellite observations and model simulations are caused only by using inconsistent variables, we also used MODIS LAI from MCD15A2Hv006 to compare with modeled LAI. This LAI product is an 8-day composite data set with a spatial resolution of 500 m [62]. It is produced using a main algorithm based on the radiative transfer model and a backup algorithm using the relationship between NDVI and LAI.

We also apply GPP from the Vegetation Photosynthesis Model [63] as a proxy of vegetation photosynthesis. This GPP data set is based on an improved light use efficiency theory and is driven by remotely sensed MODIS Enhanced Vegetation Index and climate data from NCEP Reanalysis II.

In addition, we use the NOAA Global Inventory Monitoring and Modeling System third-generation global data set (GIMMS NDVI_3g_, 1982–2018, original resolution 1/12 × 1/12° and 15-day) observed by the Very High Resolution Radiometer (AVHRR) sensors for a longer period estimation [64,65].

#### Simulations by DGVMs

We study modeled responses to drought using simulated LAI, defined as the amount of leaf area per unit of ground area. The gridded estimates of LAI from nine process-oriented DGVMs in the TRENDY-v8 model intercomparison project are used (Supplementary Table S1), including JSBACH, JULES-ES, LPJ-GUESS, LPX-Bern, ORCHIDEE-CNP, SDGVM, OCN, ISBA-CTRIP and VISIT. We apply model simulations from the TRENDY S3 experiment, which has the full set of temporally changing forcings, including CO_2_ levels, climate and land use.

#### Soil drought estimated by CMIP6 models

We select 12 ESM simulations, operated with ‘all forcings’, from the CMIP6 archive (Supplementary Table S2). These forcings are the combination of shared socio-economic pathways (SSPs) and forcing levels of the representative concentration pathway in the Scenario Model Intercomparison Project [50]. We select only the ESMs where the original spatial resolutions were ≤2° to avoid the potentially large uncertainty caused by lower resolutions. We use these multi-model climatic projections under two emission scenarios of SSP2-4.5 for intermediate emissions of air pollutants and greenhouse gases and SSP5-8.5 for high emissions.

We use the soil-moisture content for the 0- to 10-cm layer (CMIP6 variable name: ‘mrsos’) from the CMIP6 simulations to indicate future drought, mirroring our similar satellite-based measurements. All ESM variables are interpolated to a spatial resolution of 0.5° × 0.5° to match the satellite-derived drought thresholds.

## STATISTICS

### Feature extraction using PCA

Earth-observed (EO) data enable major new insights into the functioning of the land surface, offering incredible high-resolution spatial information. However, the remote sensing technique only provides satellite observations for a relatively short period (i.e. 18 years in this study). Here, we focus on detecting vegetation response to rarely occurring drought. With only 18 years of satellite observations, obtaining sufficient samples of anomalous events for statistical analysis at an individual grid is difficult and has large uncertainty. At the high spatial resolution of EO data, adjacent points are not independent and so a method is needed to identify the dominant geographical modes of variation. A method strongly suited to these requirements is PCA, as used elsewhere for feature extraction in anomalous event detection [22,66,67].

In the first step, we apply PCA-based feature extraction, such that in the low-dimensional space of principal components, similar grid cells are close to each other, even if they may be geographically distant (Step (i) in Supplementary Fig. S2). This method finds a compromise between fully local and global thresholding and refrains from (i) inaccurately estimating drought impacts on vegetation growth by setting a global unified threshold for each grid and (ii) setting an equal distribution of drought occurrence for each grid at the spatial extent against the reality that drought events do not occur equally everywhere. Using PCA, we identify areas characterized by climate and vegetation similarities, and break through the limitations for short-term satellite observation and rarely occurring anomalous events.

We select a set of variables that are believed to meet the need that (i) they impact the characteristics of drought occurrence and related vegetation growth levels and (ii) they contribute to the geographical distinction of climate conditions and vegetation distribution, composition and growth condition. Here, seven variables are used for our PCA derivation: mean annual precipitation (MAP), mean annual temperature (MAT), interannual variability of the vapor pressure deficit (VPDvar), the fraction of tree cover (Treefrac), interannual variability of NDVI and NIRv (NDVIvar and NIRvvar) and species richness (SpeciesN) (see details in Supplementary Table S3). Among them, the interannual variabilities of VPD, NDVI and NIRv are calculated as the standard deviation of variable anomalies.

MAP, MAT and VPDvar are used as climate forcing factors. MAP and MAT represent the mean climatological condition for vegetation growth. We also use VPDvar as a metric of the interannual variability of atmospheric water demand. Furthermore, we include the Treefrac variable because it provides information on land cover types and is known to characterize divergent ecosystem responses to water availability [38,67]. Biodiversity is believed to affect the response of the ecosystem to drought [68,69] so we also use SpeciesN to indicate the functional diversity and stability of the ecosystem. Finally, NDVIvar and NIRvvar are used to reflect the natural variability of vegetation greenness and photosynthesis. Note that variations of kNDVI and CSIF are not considered in our PCA analysis. We exclude kNDVI to avoid information redundancy because its variability is similar to NDVI. We also exclude CSIF due to the short record of OCO-2 SIF 2014–2017 used to reconstruct CSIF, which may not show a full representation of the interannual variability of vegetation growth. Additionally, variables that describe soil conditions such as soil fertility are not included because they did not provide improved explanatory power (Supplementary Fig. S4). This is perhaps because the impacts of soil characteristics may have been reflected in vegetation covers, the natural variability of vegetation growth and ecosystem biodiversity.

Accordingly, seven principal components (PCs) of PCA are obtained, contributed by all original variables and ranked by explained variances in decreasing order. The PCs replace the seven original variables to become new features so each grid cell possesses values of PC1–PC7. By comparing the cumulative explanation of the PCs (Supplementary Fig. S3), we chose to retain the first three PCs, as they explain, globally, 83% of the variance, yet reduce dimension and classify global lands into different clusters with comparable climate and vegetation conditions. As shown in Supplementary Fig. S3, taking the first three PCs substantially increases the cumulative variance explained (compared with taking the first two PCs (66%) or one PC (42%)) and allows the distinguishing of feature differentiation of global lands driven by vegetation cover and climates.

In the next step, we link a target grid cell *x(lat, lon)* in the geographical domain and find its location point *p(u, v, m)* in the 3D space of PC1–PC3, based on their first three PC scores (Supplementary Fig. S2). Taking *p(u, v, m)* as a center in the PC space, we find all the neighbor points in a window of 3 × 3 × 3 meshes whose width is within 4% of the total length of the corresponding PCs [22]. Although *p(u, v, m)* may not be geographically close to the grid cells that are located as the neighbor points in the 3D PC space, they are divided into the same clustering based on comparable climate and vegetation conditions. In such a PCA clustering (i.e. all points in a set of 3 × 3 × 3 meshes), we define droughts (<10th percentile for anomalies surface or root-zone soil water content) and vegetation response to drought (<10th percentile for anomalies of vegetation indices). For example, if one clustering has 50 points (point *p* + 49 neighbor points), the anomalies of the 10th percentile will be derived based on 900 values (50 grid cells × 18 years) of drought or vegetation indicators. We then have available the coincidence rates, as well as the drought threshold based on those 50 grid cells in the clustering, and the final results are assigned to the grid cell *x(lat, lon).*

Here, splitting up the space of PCs allows us to obtain more regionally relevant event thresholds (for details, see [22]) and achieve the compromise between a completely global scale and overly local scales. The global map of drought thresholds in this study can reflect the spatial heterogeneity because our approach to deriving thresholds retains more information than those for the regional scale. On average, there are only ∼0.2% of all grid cells located in the same centered mesh of a 3 × 3 × 3 window in the space of the PCs so that these grid cells would be assigned by the same threshold values (see the spatial pattern (b) in the diagram, Supplementary Fig. S2). This ensures that different grid cells would lose less information on differentiating the divergent vegetation responses to drought.

### Response trajectories for vegetation to different levels of drought stress

#### Coincidence analysis

The main overall feature of our calculations is coincidence analysis [21] to identify droughts and the resulting suppression of vegetation greenness and photosynthetic activity. In particular, this method allows us to build response curves for vegetation activity at different levels of drought stress. We assume vegetation responses to be independent between years and including when major drought happens in consecutive years. That is, we assume no interannual memory that may cause lagged effects as well as post-drought legacy effects. For all grid cells in each moving 3 × 3 × 3 set based on the first three leading PCs (see details above), we identify drought occurrence indicated by SMsurf anomalies and vegetation response indicated by anomalies of different satellite observations or model simulations.

We quantify the coincidence rate (*r*) using the single vegetation threshold of any fraction <10th percentile of vegetation anomaly (${\theta }_{veg < 10th}$). We applied <10th percentile to define vegetation response to drought because, at this level, the impacts of drought on vegetation have widely occurred and can be detected and assessed in different locations under current climate change [20]. We suggest that any drought frequently crossing this threshold would accumulatively impact the vegetation development and may provide an important insight into the potential change of ecosystem structure and functioning under future climate change. The crossing of this threshold is recorded per drought year when the drought level of the corresponding growing seasons is equal to or less than the percentile-based anomalies (${\theta }_{dro \le q}$) of SMsurf. Hence, *r* is calculated as:


(1) \begin{eqnarray*} r = \frac{{\textit{Fre}(G < {\theta }_{veg < 10th}\forall t{\,\,\rm{ when }}\,\,{\theta }_{dro} \le {\theta }_{dro \le q})}}{{\textit{Fre}(\forall t{\,\,\rm{ when }}\,\,{\theta }_{dro} \le {\theta }_{dro \le q})}}, \end{eqnarray*}

where *G* is a metric of anomalous vegetation greenness or photosynthetic capacity, *t* is the drought year and ${\theta }_{dro \le q}$ is a soil-moisture threshold expressed by percentile *q*. The denominator of Equation (1) is the frequency of drought occurrence below percentile *q*. The numerator is similarly the frequency of when the soil moisture is below percentile *q*, except we only now count this time when additionally there is vegetation suppression.

Here, the drought threshold *q* corresponds to the anomalies of SMsurf as a function of the percentile ranges on the *x*-axis in Supplementary Fig. S1. Smaller *q*-values correspond to more extreme soil-moisture deficits. Quantity *r* for each location accordingly corresponds to the *y*-axis in Supplementary Fig. S1 (and subsequent plots). In each window, *r* is estimated only when sufficient samples (>162, 9 grid cells × 18 years) are available.

#### The relationship between *r* and *q*

We explore the relationship between percentile-based drought thresholds and the coincidence rates, *r*, of drought-vegetation anomalies. It is expected that the coincidence rates of concurrence between drought and vegetation response are low and change little when experiencing slight-to-mild droughts owing to the resistance of the vegetation (Phase A in Supplementary Fig. S1). The coincidence rates then sharply increase once the drought stress levels exceed their tolerance limits (Phase B). To confirm this form of response, we sampled *r* and ${\theta }_{dro \le q}$ with *q* ranging from the 1st to 50th percentiles (with an interval of 1 percentile) of soil-moisture anomalies. By testing different non-linear curve fitting, we believe an exponential-type curve is the optimal functional form to capture the relationship between drought threshold and drought-vegetation-anomaly coincidence rates (see details in the Supplementary Discussion 2 and Supplementary Figs S15 and S16). We thus applied an exponential function across the full range of 1st–50th percentiles, written as $r = m{e}^{\beta {\theta }_{dro \le q}} + k$, to test whether the response trajectories of vegetation follow a generic response curve that a flexible non-linear fit is better than a linear fit. If so, the relationship between drought stress levels and vegetation decrease shows threshold behavior and vegetation response can be distinguished from two segments. Hence, a breakpoint is able to be searched.

We exclude areas where response trajectories of vegetation do not follow a generic format of the response curve if (i) a simple linear model outperforms a non-linear model. To select the better model, we apply the Akaike information criterion (AIC) to evaluate the fitness of linear and non-linear models. If AIC of the linear model is larger than that of the non-linear model unless the linear fitness performance is superior (*R*^2^ > 0.95), we regard the response curve follows non-linear relationship between drought severity and vegetation response. (ii) Poor fitness of the exponential function for the response curve (the adjusted *R*-squared ≤ 0.5) (Supplementary Fig. S6): in order to avoid mistakenly identifying inflection points from satellite data, we consider thresholds in the areas where the maximum value *r* is <0.3 (Supplementary Fig. S10) or thresholds detected only in a single vegetation proxy possess (not marked by dots in Fig. 2) are unrobust.

### Identification of drought thresholds for vegetation response

We identify the percentile-based drought threshold (*T_SMsurf_*) values, which characterize the initiation of vegetation response to rising drought levels. *T_SMsurf_* are percentile values corresponding to the anomalous soil-moisture ${\theta }_{dro \le {T}_{SMsurf}}$. Although we fit a function for *r* with an exponential continuous in its first derivation, here we search for *T_SMsurf_* as an inflection point of an abrupt change by vegetation as drought intensifies. We use a piecewise linear regression [23,70] to obtain two distinct linear segments based on the samples *r* with ${\theta }_{dro \le q}$ when *q* = 50th, 49th, 48th, …, 3th, 2th, 1th of SMsurf:


(2) \begin{eqnarray*} r = \left\{ \begin{array}{l}{\beta }_0 + {\beta }_1{\theta }_{dro \le q} + \varepsilon \\ {\rm{ for }\quad }{\theta }_{dro \le q} \ge {\theta }_{dro \le {T}_{SMsurf}}\\ {\beta }_0 + {\beta }_1{\theta }_{dro \le q} + {\beta }_2({\theta }_{dro \le q} - {\theta }_{dro \le {T}_{SMsurf}}) + \varepsilon , \\ {\rm{ for }\quad }{\theta }_{dro \le q} < {\theta }_{dro \le {T}_{SMsurf}} \end{array} \right.\!\!\!\!\!\!\!\!\\ \end{eqnarray*}

where ${\theta }_{dro \le {T}_{SMsurf}}$ is the inflection point of the two-segmented linear regression between ${\theta }_{dro \le q}$ and *r*; ${\beta }_0$, ${\beta }_1$ and ${\beta }_2$ are fitted regression coefficients of the two distinct linear segments, from which ${\theta }_{dro \le {T}_{SMsurf}}$ is inferred and $\varepsilon $ is the residual of the fit. Note that ${\beta }_1$ is non-zero when ${\theta }_{dro \le q}$ is <${\theta }_{dro \le {T}_{SMsurf}}$. The optimal segmentation with a best fit was determined by the minimal square error of this linear model. To minimize the possibility of incorrectly identifying *T_SMsurf_*, we exclude cases in which the slope (${\beta }_1$) of the first segment of the line regression (the upper line in Equation (2)) is larger than the slope of the second segment (the lower line in Equation (2)) to avoid deriving the wrong inflection point. We present the sample locations for estimating percentile-based thresholds in Supplementary Fig. S5.

Using surface soil moisture, the global patterns of *T_SMsurf_* based on NDVI, kNDVI, NIRv and SIF verify the consistency among the vegetation indicators (Supplementary Fig. S7). We thus average drought thresholds across all satellite observations at each grid and present histograms of all data combinations (Fig. 2a). To evaluate the impacts of drought durations on the thresholds, we also detect the *T_SMsurf_* for each month (Supplementary Fig. S12) similarly to Fig. 2a. Mean *T_SMsurf_* values are averaged by the *T_SMsurf_* of January to December only if those months are in the growing season for that location. We make a comparison between the average monthly *T_SMsurf_* for months during the growing season only and the whole growing-season *T_SMsurf_*.

For the model evaluation of *T_SMsurf_*, we exclude the model simulations from JULES-ES, ORCHIDEE-CNP and SDGVM. Among all nine vegetation models (Supplementary Table S2), JULES-ES, ORCHIDEE-CNP and SDGVM have very low coincidence rates (maximum values < 0.3) for >90% of the grid cells (Supplementary Fig. S17). We thus consider JULES-ES, ORCHIDEE-CNP and SDGVM as having failed to capture the spatial patterns of thresholds. To further investigate the difference between observation-based thresholds and model-derived thresholds, we also display the drought thresholds along the gradients of aridity index and tree cover for areas overlapped by both observation-derived and model-derived thresholds (Fig. 3). We exclude those meshes where averages are calculated by thresholds from fewer than five grid cells in Fig. 3.

### Shuffling test to remove randomness

We perform a statistical ‘shuffle’ test to assess the significance of the critical thresholds for testing the robustness of our findings. We create 500 surrogate time series of the vegetation time series by randomly shuffling the original dates of the vegetation time series, and estimate the *T_SMsurf_* based on the time series of the drought indicators and the surrogate vegetation time series. Our null hypothesis is that *T_SMsurf_* for the original and surrogate vegetation time series do not differ systematically. This hypothesis applies to the ‘non-significant *T_SMsurf_*’. An alternative hypothesis is that the thresholds *T* for the original and surrogate vegetation time series differed systematically, and so our discovered links between driver and response are valid. The estimates of *T_SMsurf_* are assumed to reject the null hypothesis and be significant if they were 2.5%–97.5% outside the surrogate distribution, corresponding to *P* < 0.05. When we perform such an analysis, we find we reject the non-significant *T_SMsurf_* (*P* > 0.05) possibility and so adopt the alternative hypothesis that there is a non-linear relationship, as based on both satellite observations and model simulations from DGVMs.

### Assessing future changes in *T_SMsurf_*-inferred drought frequencies

For each grid, we first derive anomalies of the moisture content of surface soil for these two periods by subtracting their linear trends of 2001–20 and 2081–2100, respectively. We then derive the anomalies of soil moisture corresponding to the percentiles of satellite-derived *T_SMsurf_* and calculate the probabilities of the occurrence of droughts smaller than *T_SMsurf_*-based anomalies at historical (2001–20) and future (2081–2100) times. Since model simulations are suggested to have a poor characterization of drought-related physiological mechanisms (Fig. 3c), we only predict the changes in probabilities of drought occurrence along the future aridity index. Considering that those satellite-derived *T_SMsurf_* values still maintain large uncertainty in some tropical, boreal and Arctic areas, we exclude these areas when predicting the future probability of drought occurrence.

## DATA AVAILABILITY

MOD13C2 is available at https://lpdaac.usgs.gov/products/mod13c2v006/; MCD43C4 is available at https://lpdaac.usgs.gov/products/mcd43c4v006/; MCD15A2H Version 6 is available at https://lpdaac.usgs.gov/products/mcd15a2hv006/; CSIF is available at https://osf.io/8xqy6/; GLEAM soil moisture is available at https://www.gleam.eu/; Model simulations of CMIP6 is available at https://pcmdi.llnl.gov/CMIP6/.

All computer codes for the analysis of the data are available from the corresponding author upon reasonable request.

## Supplementary Material

nwad049_Supplemental_Files
