# Supplementary material for: Global variations in critical drought thresholds that impact vegetation
Source: Natl Sci Rev. 2023 Feb 24;10(5):nwad049. doi: 10.1093/nsr/nwad049 (PMC10103823; doi:10.1093/nsr/nwad049)
Supplement: nwad049_Supplemental_Files [file nwad049_supplemental_files.zip › Supp_File_drought_threshold_nwad049.docx]

***Supplementary Information of***

**Global variations in critical drought thresholds that impact vegetation**

**Supplementary Discussion**

1. **Sample locations for percentile-based thresholds.**

We randomly select five sample locations (L1-L5) with different vegetation types and climatic backgrounds (see Supplementary Fig.5) to illustrate observed vegetation NDVI responses to different levels of drought stress based on the 1-50th of percentiles for soil water deficit. L1-L5 are covered by tropical forest, tropical savanna, temperate cropland, arid shrubland, and arid grassland, respectively. They illustrate that vegetation have the consistent formats in response to drought regardless of the climate backgrounds and vegetation covers (Fig. S5c). But different performances of those sample locations also suggest that vegetation have different capacities to resist drought.

L1 is featured by both high temperature (~25℃) and high mean annual precipitation (~1900 mm y^-1^) (Supplementary Fig.5b). It illustrates the case that tropical forest was highly drought tolerant so it has quite low coincidence rate of drought-vegetation anomalies at slight and moderate drought levels (phase A). This sample location thus has a drought threshold *T_SMsurf_* corresponding to the relative high soil moisture deficit (8.3th ± 1.7 for L1), followed by a narrow gradient of responses to droughts beyond *T_SMsurf_*. This location covered by forest is thus suggested to quickly collapse once the drought stress level exceeding their tolerance limitation. L2 are featured by lower mean annual precipitation (1700 mm y^-1^) than L1 and similarly high temperatures (~25 °C). It illustrates the case where the vegetation is more vulnerable to drought than L1.

By comparison to L1 and L2, although L3 covered by cropland with wet and hot climates (900 mm y^-1^ of mean annual precipitation and ~25℃ of mean annual temperature) has lower resistance to drought, suggested by a higher coincidence rate for response at phase A and smaller percentiles for drought thresholds (*T_SMsurf_* =14.5th of soil moisture deficit for cropland).

L4 and L5 illustrate two different cases for vegetation response to drought. L4 is prone to be impacted by drought, featured by very dry condition (~380 mm y^-1^ of precipitation) but high temperature (~26℃). The relatively low percentile of drought threshold for L4 (9.4th ± 1.6) suggests that woody plants (shrubland for L4) are highly adaptive and resistant to drought. In comparison, L5 is featured by both low mean annual precipitation (~390 mm yr^-1^) and low temperature (~8℃). The development of grass growth in L5 is thus limited by both temperature than water availability, and illustrating higher sensitivity to drought (*T_SMsurf_* =11.6th ± 2.4).

1. **Model selection for curve fitting**

In our study, we define drought based on the percentile approach, which highlights the rare drought frequency. This definition ensures that the identified drought events match the current regional climates, and we are able to detect equal magnitudes of drought events with one unit of percentile change. Based on this percentile-based approach, the relationship between drought threshold (*q*) and drought-vegetation-anomaly coincidence rate (*r*) follows exponential laws. Taking the grid cell located at (60°N, 160.5°W) as an example (Supplementary Fig. 15a), we fit the curve using three functions, an exponential function, a logistic function with a linear term and a logistic function with linear and quadratic terms. The results show that the exponential function (the red curve) has higher R^2^ value than logistic functions (blue and purple curves). Besides, by testing different levels of vegetation suppression, the exponential-type curves are robustly fitted (9.0 °N, 66.5°W, Supplementary Fig. 16a).

In addition to defining drought using the percentile-based approach, the standard deviation approach, which highlights the changing severity of drought stress, is also widely used in many studies. Herein we use the same grid cell (60°N, 160.5°W, Fig. S5b) as an example to confirm the nonlinear relationship between drought stress and vegetation response does exist, irrespective of different methods. In Supplementary Fig.15b, we use severity of soil drought (quantified based on the standard deviation approach) as an independent variable. It is suggested that, with the increasingly negative extreme of soil moisture, the coincidence rates rapidly increase and reach the maximum value, i.e. r=1.0. After that, even if the drought severity becomes higher, r would be 1.0. Therefore, the response curve based on the standard deviation method is in line with the percentile method. Using an example from another grid cell (Supplementary Fig.16b), we see that even defining different levels of vegetation suppression does not change the non-linear relationship (Supplementary Fig.16b).

1. **The uncertainty and limitations**

In this study, we build a framework for detecting drought thresholds based on theoretical understanding, merged with measurements. To derive robust results, we apply data from multiple sources and perform statistical tests at different steps to eliminate the impacts of uncertainty as much as possible. However, some limitations and uncertainties inevitably exist in processing of observations, drought estimation, and climate model projections of future change. We outline some of these limitations and uncertainties.

***(i) Uncertainty in estimating the drought thresholds***

First, the hydrometeorological data and remotely sensed vegetation proxies have different original resolutions, so some small bias is caused in the procedure to unify the resolutions to a common grid of 0.5×0.5 degrees.

Second, based on the principal component analysis, we estimate the coincidence rate and drought threshold of one grid cell within 3×3×3 meshes along the first three PC axes, which allows us to make more general statement than possible at single points yet retain geographical information that would be unavailable with a single global scaling. Although we believe that our results capture the local performance of the response of vegetation, the choice of 3×3×3 meshes based on the PCA can introduce some uncertainty. The limited time scales due to length of satellite data also contributes uncertainty. Additionally, as shown in Extended Data Fig. 10, drought thresholds tend to have higher percentiles when estimated for individual months within the growing season than when considering the growing season as a whole (Fig. 2a). Therefore, the whole-growing-season-derived drought thresholds in our main finding might have some uncertainty caused by either different drought durations or divergent vegetation responses to drought within the growing season. Further work looking at monthly disaggregation is required in the future.

Moreover, the values of different vegetation proxies (i.e. NDVI, kNDVI, NIRv, and SIF) reflect a range of biophysical or biochemical features of vegetation, including different aspects of foliage cover, canopy structure, and photosynthesis activity. As there may be different timings of physiological processes operating for biome responses to drought, this too will cause uncertainty. In addition, the data quality of the satellite data differs between regions. For example, in tropical regions, we reiterate that there may be large uncertainty in satellite-based estimates of vegetation response due to data issues including atmospheric effect, saturation phenomenon, and sensor factors like the sun-sensor geometry. Indeed, we verify that there exists a much larger uncertainty of drought thresholds in tropical and subtropical regions for different vegetation proxies (Supplementary Figs. 6-7).

In addition, we estimate the drought thresholds without considering lagged responses of vegetation to drought. This practice simplifies the complex process of vegetation response to drought but could lead to underestimation of drought thresholds, especially in dryland regions with longer response lag and for forests with strong resistance.

***(ii) Uncertainty in predicting future drought risks***

For future projections of drought occurrence, there is also some uncertainty such that we need to interpret our results with some caution. First, the selection of the metric, of soil moisture has a strong effect on estimated future changed probabilities. The change in soil moisture change may not fully represent realistic vegetation water stress, due to uncertainties in the depth or spread of roots, as well as the divergent stomatal performance of different biomes. We follow standard practice and predict the future change of soil moisture deficit using the soil moisture content for the 0-10 cm layer in the CMIP6 simulations. Although we find very similar patterns of observed-derived drought thresholds using surface soil moisture and root-zone soil moisture using GLEAM data (Fig. 2 and Supplementary Fig. 18), there may be a different balance between future changes of moisture in the surface and deep soil. For instance, under a substantially warmer climate, moisture would be reallocated away from the surface more readily through increased evapotranspiration, so a more severe water deficit may be found more in the surface layer than the root-zone layer (Cook et al., 2020).

Second, given our noted inaccuracies in reproducing observed drought thresholds in global vegetation models, we instead aim to establish drought thresholds for vegetation based on model-led estimates of climatological change only, merged with data-led estimates of ecosystem thresholds. When using this approach to predict future changes, this implicitly assumes relatively little vegetation acclimation to future climate change. Yet rising atmospheric CO_2_ concentration may partly ameliorate the impacts of future drought stress by increasing carbon gain per unit of water loss, thus partially offsetting any increases in crossing drought thresholds. The magnitude and persistence of this carbon efficiency effect has remained controversial so far under combined rising temperature and elevated CO_2_. In addition, our data-led estimates of thresholds are derived with an assumption of no legacy effects exist (e.g., interseasonal or interannual memory). However, previous studies report that the effects of the drought legacy can impact post-drought recovery of vegetation (Anderegg et al., 2011; Huang et al., 2018). The legacy effect of drought could impose accumulative negative impacts on vegetation should there be more frequent drought events in a changing climate, and alter vegetation response to drought. The difference in drought thresholds estimated in a longer period 1982-2018 for GIMMS NDVI_3g_ compared to 2001-2018 for our four vegetation indicators (Fig. 2a vs. Supplementary Fig. S11c) provides some support for the change in thresholds.

Therefore, to advance beyond our approach that derives data-based drought thresholds (and is thus independent of DGVMs), there is an urgent need for process models projecting future drought risks for vegetation in a changing global environment. This activity will require the use of DGVM capable of accurately estimating drought thresholds and including their evolution as CO_2_ increases. Our approach provides a very powerful benchmark of the capability of the next generation of DGVMs to project such drought thresholds, at least for current climatic conditions.

**Supplementary Figures**


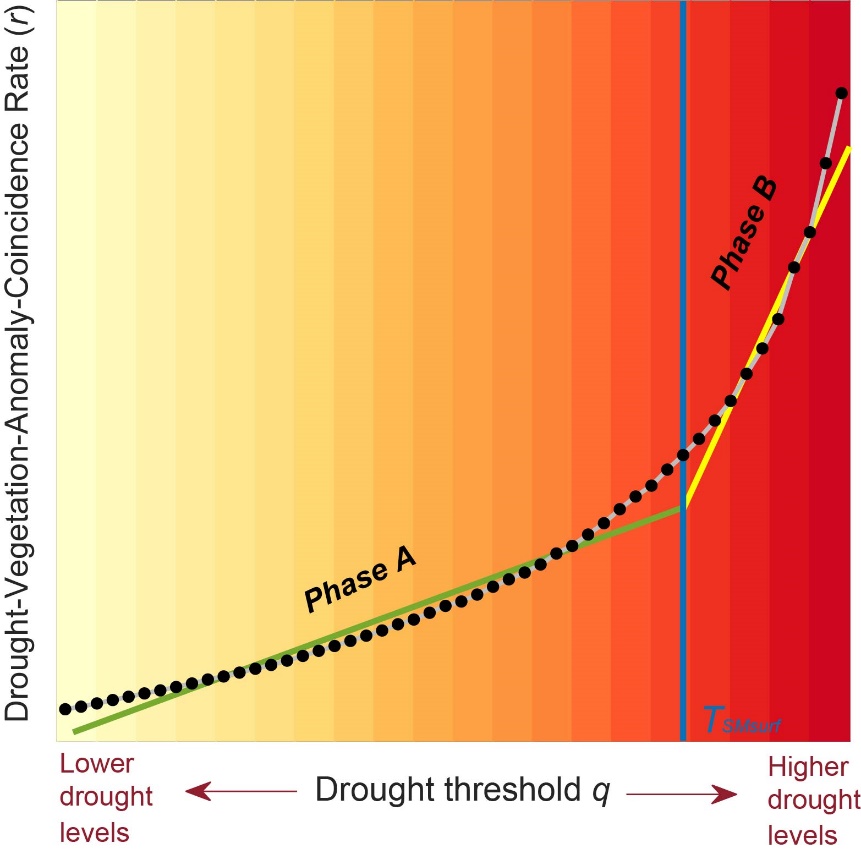


**Supplementary Figure 1. Conceptual diagram of drought-vegetation-anomaly-coincidence rate with increasing percentile-based drought thresholds.** The panel is derived by considering all years when drought is equal to or smaller *q* for drought stress on the *x*-axis. *q* is a certain threshold in 1^st^ - 50^th^ percentile ranges for the distribution of soil moisture anomalies. For different drought levels, recorded on the *y*-axis is the coincidence rate between drought occurrence and vegetation greenness or photosynthetic activity suppress by 10^th^ of vegetation anomalies. The value of a coincidence rate on the *y*-axis is unitless. *T_SMsurf_* is an inflection point for coincidence rates of vegetation response to surface-soil-moisture drought. The optimal segmentation with the best fit was determined by the minimal standard error of linear models. Phases A and B are two different stages for vegetation response defined in the main text.


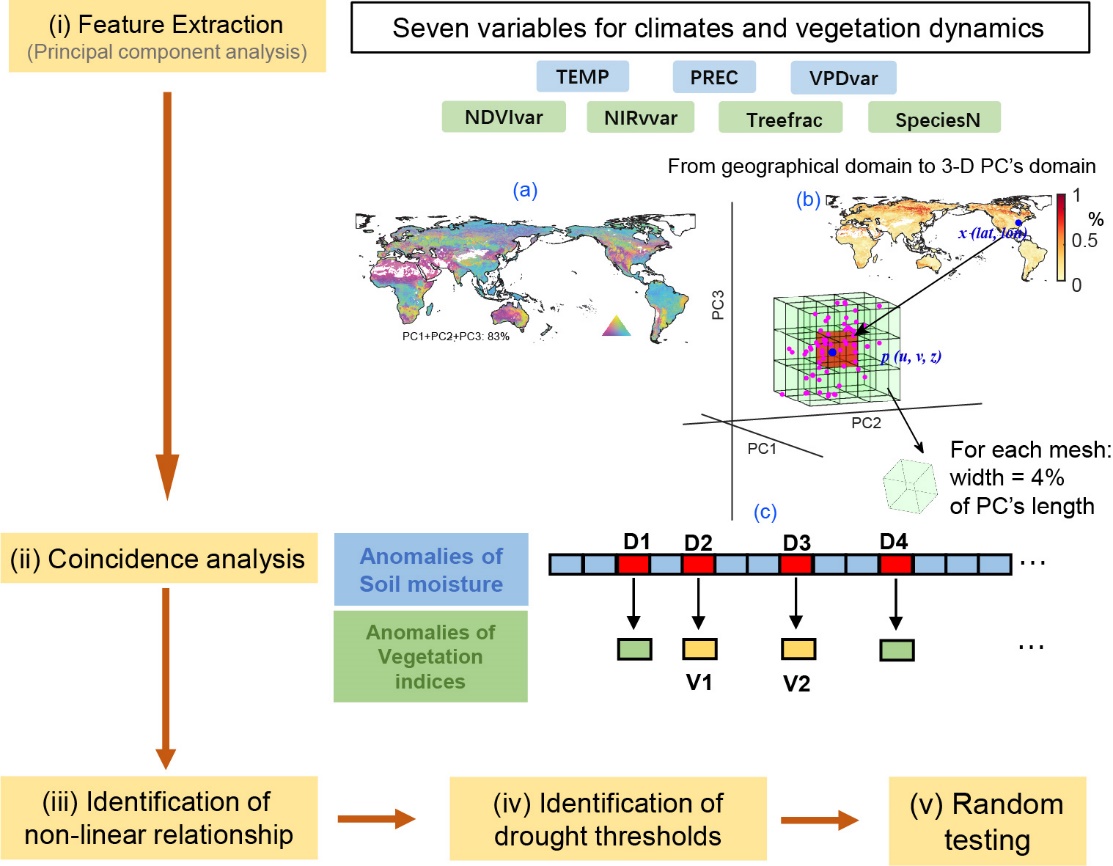


**Supplementary Figure 2.** **Illustration of the procedures for identifying drought thresholds**. (i) Extracting land features around the world is based on seven forcings and achieved through principal component analysis (PCA). We change each grid from the geographical space with latitude and longitude information to 3-D domains in principal component (PC) space (using the first leading PC1-PC3). The grid is located in the centre mesh (red) of a 3×3×3 meshes moving window. The width of each mesh is 4% of the PC’s length (maximum-minimum PC scores). The global map (a) shows the geographical space of PC1-PC3 (as shown in Figure S4b) and the spatial pattern (b) shows the local percentage of grid cells that each grid shares the same threshold values with them on global lands. (ii) Based on this PCA-based pooling, the coincidence analysis (c) is designed to find drought years (D1, D2, D3, D4…, coloured by red) and the corresponding years when vegetation responds to drought (V1, V2,…, coloured by yellow; years with no response coloured by green). (iii) We then identify the locations where non-linear relationships hold (Fig. S15) and (iv) identifying the drought thresholds in these areas. (v) We test the robustness of the thresholds with randomness by 500 times randomly shuffling the original dates of the vegetation time series. Thresholds within the 95% distribution of all values derived from surrogate time series of vegetation proxies are removed (*p* < 0.05).

**
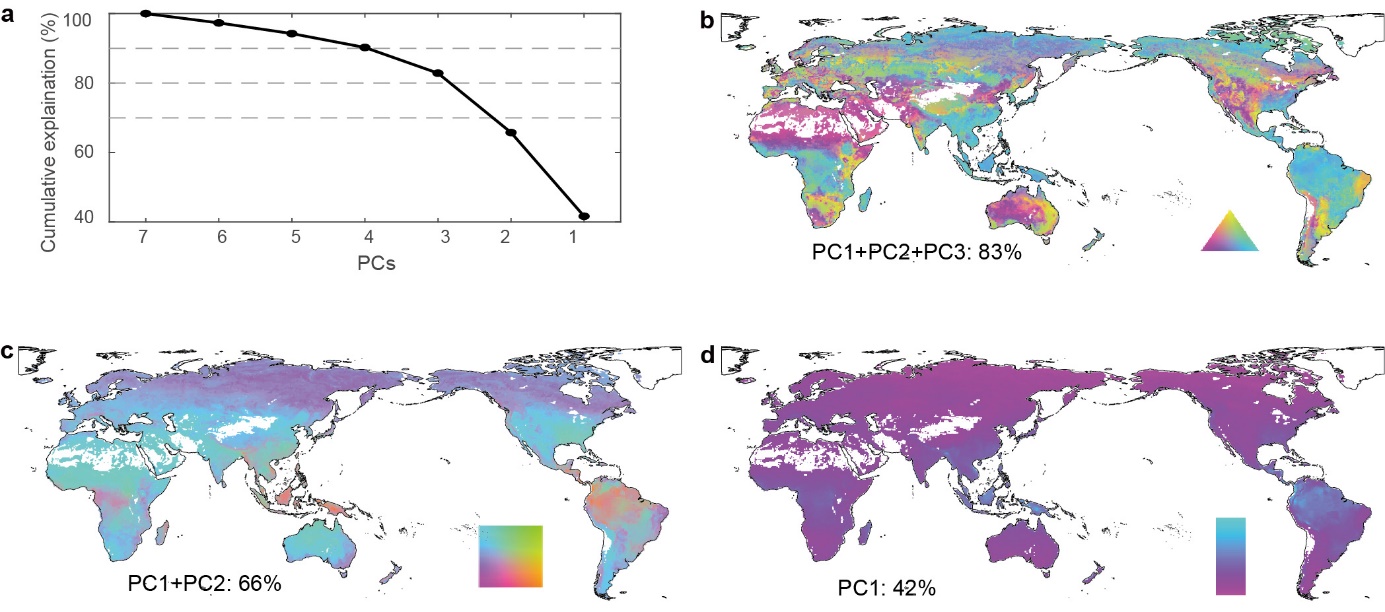
**

**Supplementary Figure 3.** **The spatial segmentation of global lands through Principal Component Analysis (PCA).** (a) The cumulative explanations of the first to seven principal components (PCs) for the seven variables MAT, MAP, VPDvar, NDVIvar, NIRvvar, Treefrac, and SpeciesN. (b) The global map of the first three principal components (PCs) cumulatively explains 83% of the variance. The colors in the three-way triangle show the different dominance of the PC in each location. (c) The global map of the first two PCs, cumulatively explains 66% of the variance. (d) The global map of the first PC explains 42% of the variance. More similar RGB colours indicate areas with more comparable grids. Seven variables used in the PCA include mean annual precipitation (MAP), mean annual temperature (MAT), interannual variability of VPD, tree cover, interannual variability of NDVI and NIRv, and species richness (see Data and Methods)


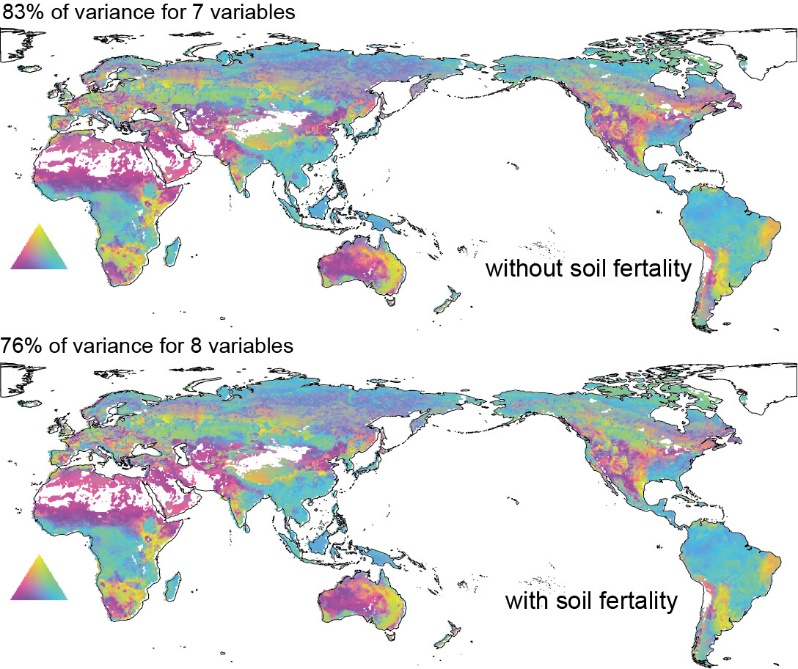


**Supplementary Figure 4. Comparison of spatial maps of the first 3 PCs without and with inclusion of the factor of soil fertility.** The figure illustrates that this variable is likely redundant, as its inclusion actually causes a weaker fit.

**
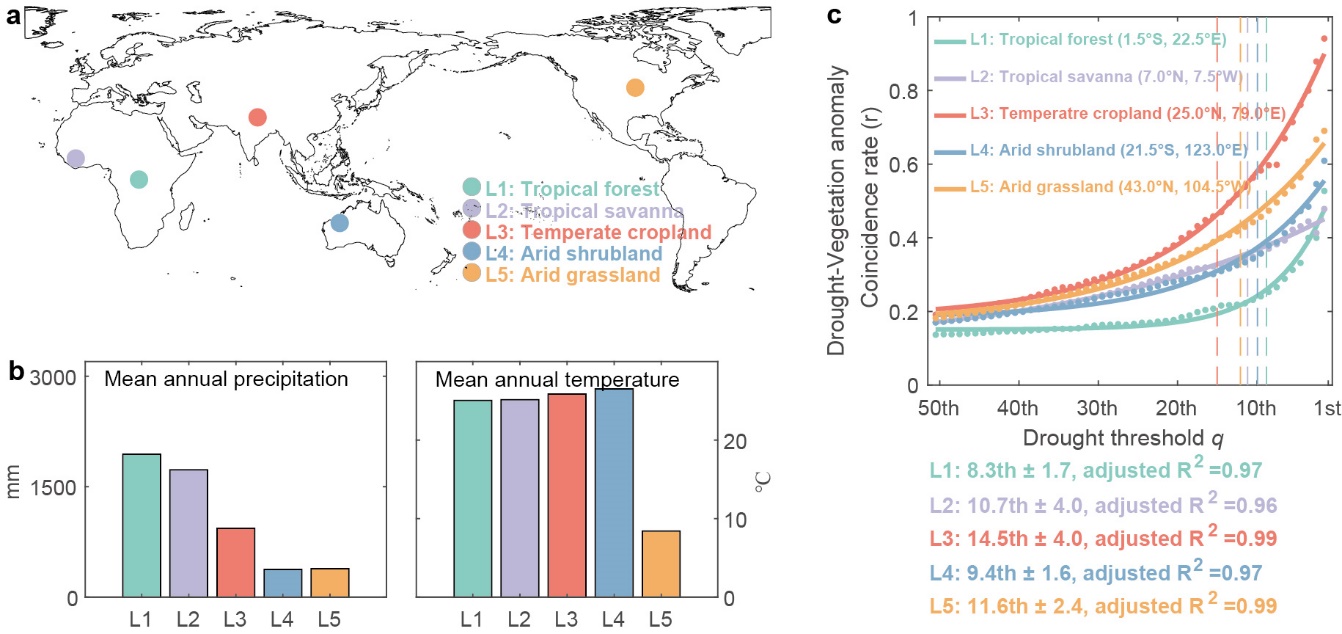
**

**Supplementary Figure 5.** **Example points for identifying drought thresholds.** **a.** Spatial distributions of sample locations L1-L5. L1, 1.5°S, 22.5°E; L2, 7.0°N, 7.5°W; L3, 25.0°N, 79.0°E; L4, 21.5°S, 123.0°E and L5, 43.0°N, 104.5°E. The information for the vegetation types in a is derived from the MODIS MCD12C1 Version 6 data product. **b.** Mean annual precipitation and temperature for sample locations L1-L5. **c.** Example locations in a for observed vegetation NDVI responses to increasing drought thresholds using percentile ranges of the surface soil moisture (SMsurf) anomalies during the growing season. For each location and drought threshold (*x*-axis), the vegetation response is defined as the probability that NDVI has decreased by <10th of NDVI anomalies. The dots show the probabilities of the NDVI response to increasing drought thresholds, from 1-50th of SMsurf anomalies, corresponding to different levels of soil water deficits. Dots of the same color are for individual locations as indicated, and the expected response curves have a form of the exponential function. The drought thresholds for L1-L5 were marked by dashed vertical lines. The texts at the right of the panel displayed the adjusted R square of the expected response curve, the percentiles of the SMsurf anomalies for the inflection points of the samples as well as the uncertainty of percentiles estimated by 1000 times bootstrapping.

**
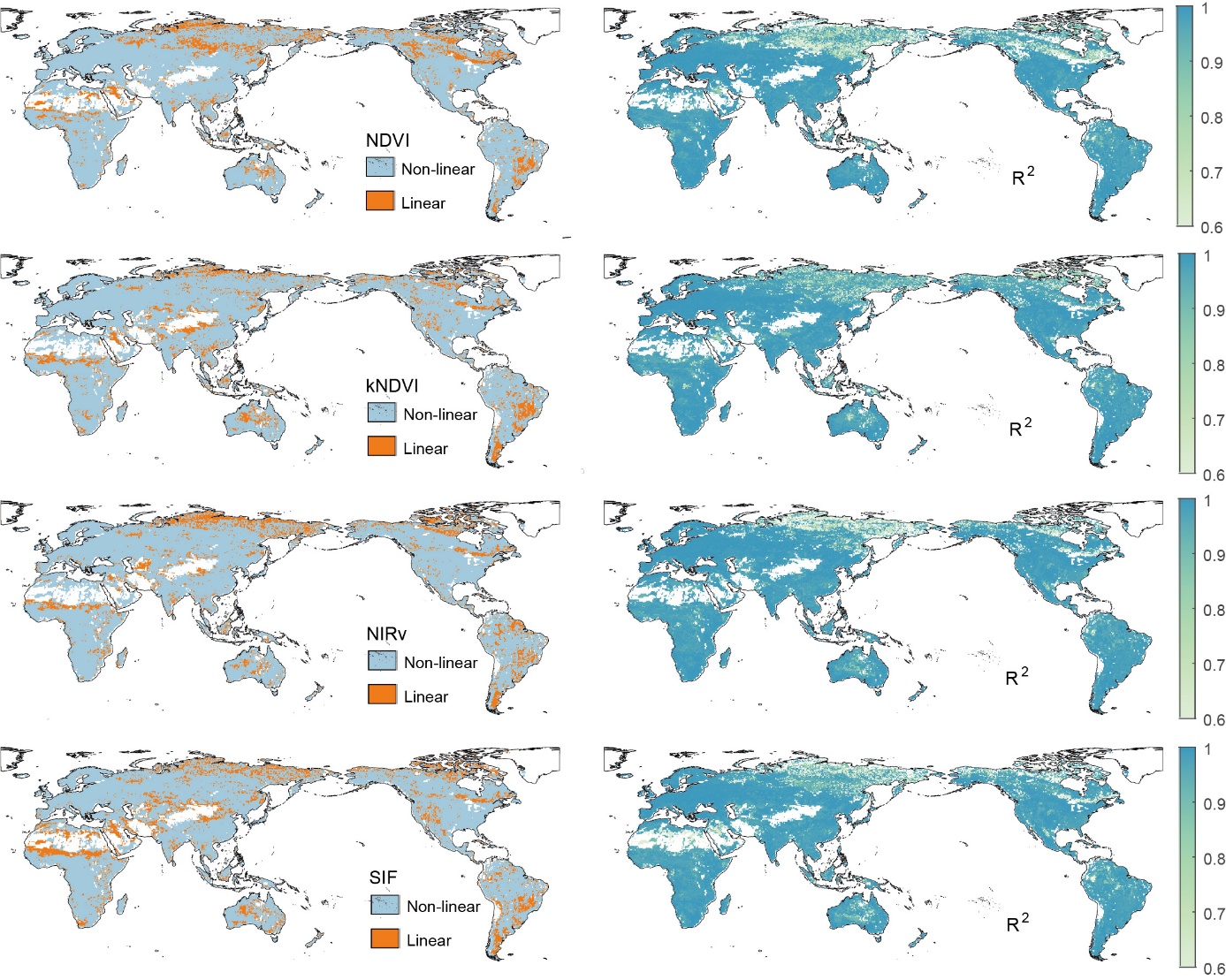
**

**Supplementary Figure 6. Global maps of regional response curves and their *R*-square of the fitted model**. The left-hand columns show where the “Non-linear” (blue) exponential response curve provides a better fit than the linear function, while “Linear” (orange) represents a response curve following a better fitness of the linear function than the exponential function. The right-hand columns are the R^2^ fit values (of the best model, nonlinear or linear, depending on location). The response curves are assessed by the surface soil moisture and four vegetation indicators NDVI, kNDVI, NIRv, and SIF, as marked by the legend.


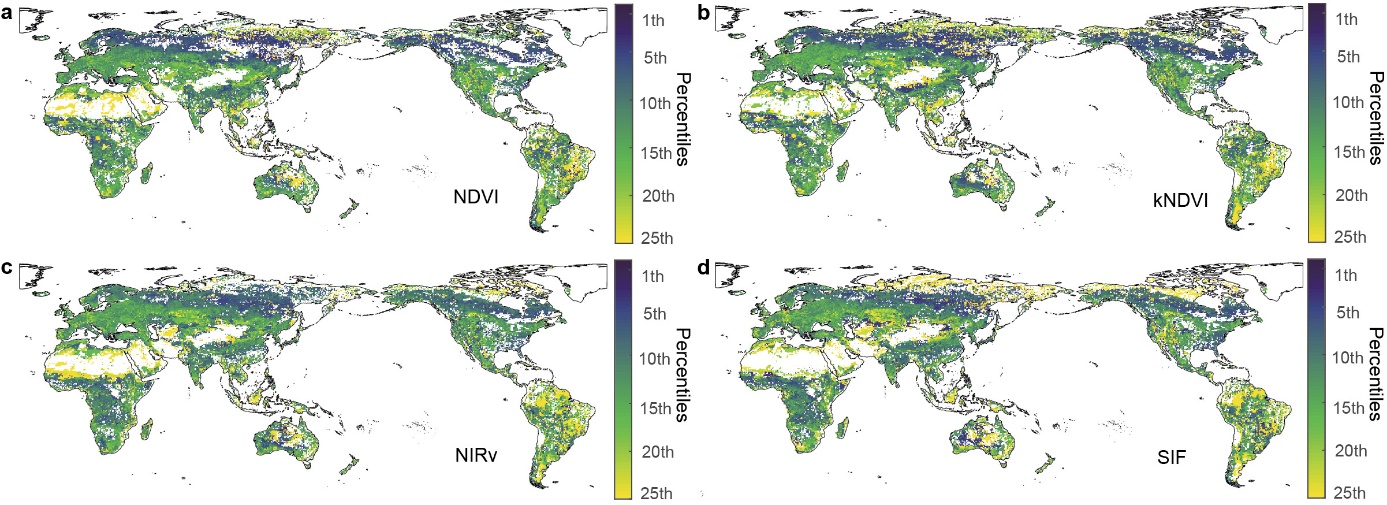


**Supplementary Figure 7. Spatial patterns of drought threshold (T*_SMsurf_*) for satellite observations (NDVI, kNDVI, NIRv and SIF) for 2001-2018.** Droughts are identified by anomalies of SMsurf. Vegetation response is defined as anomalies of the vegetation indicators (NDVI, kNDVI, NIRv and SIF) < 10th percentile. Smaller drought thresholds correspond to defining more extreme drought condition as drought.


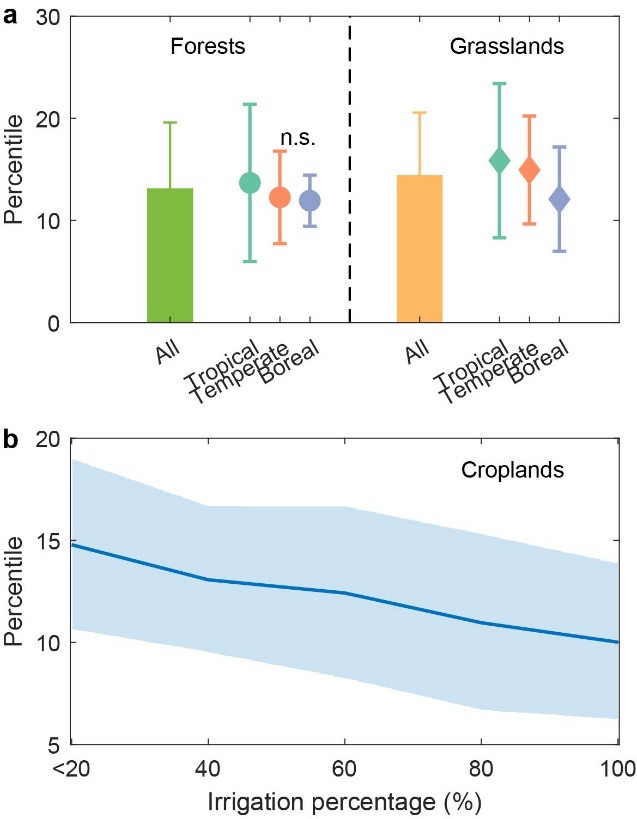


**Supplementary Figure 8. Divergence in drought thresholds for different biomes. a.** Drought thresholds of forests and grasslands. The bar charts with errorbars show the mean and standard errors for gridboxes that are either all forests (green) and or all grasslands (orange). The filled circles with errorbars represent the drought thresholds of forests from tropical (light green), temperate (red) and boreal (purple) areas. The filled diamonds with errorbars represent the drought thresholds of grasslands from tropical (light green), temperate (red) and boreal (purple) areas. For the same biome in different climates, tropical locations are statistically (*p* < 0.05) higher than temperate and boreal climates for both forest and grass, but this is not the case for forest in temperate and boreal climates (marked by n.s.). **b.** Drought thresholds vary with increasing percentages of land area equipped for irrigation. The information on land cover and climate classification (panel a) is derived from the MOD12C1 the International Geosphere Biosphere Programme (IGBP) global vegetation classification schemes and the world map of Köppen-Geiger climate classification. The irrigation information (panel b) is derived from the version 5 global map of irrigation areas developed by FAO’s Global Information System on Water and Agriculture (AQUASTAT) programme.

**
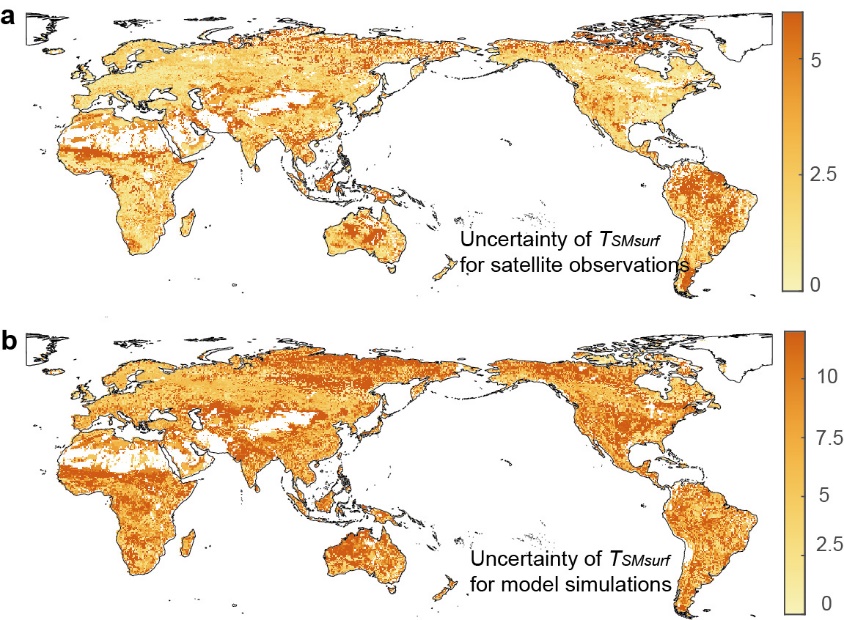
**

**Supplementary Figure 9. Spatial patterns of uncertainty in thresholds for different vegetation proxies.** (a) Uncertainty in thresholds for NDVI, kNDVI, NIRv, and SIF during 2001-2018, as estimated by the standard deviations of the four satellite observations. The color bars show the percentile values. (b) The same as a but for six simulations of DGVMs (JSBACH, LPJ-GUESS, LPX-Bern, OCN, ISBA-CTRIP, and VISIT).

**
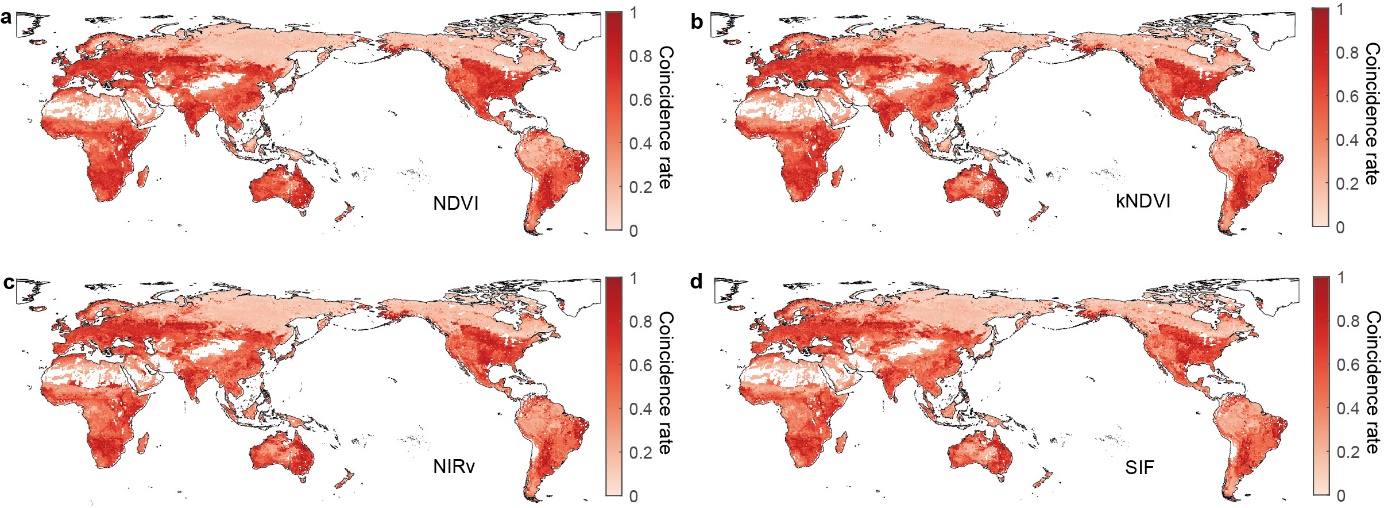
 Supplementary Figure 10. The spatial patterns of maximum coincidence rates using all testing drought stress levels (1^st^ -50^th^ percentile) during 2001-2018**. The vegetation proxies are (a) NDVI, (b) kNDVI, (c) NIRv, and (d) SIF. Droughts are identified by anomalies of SMsurf and vegetation response is defined as < 10th percentile of vegetation anomalies.


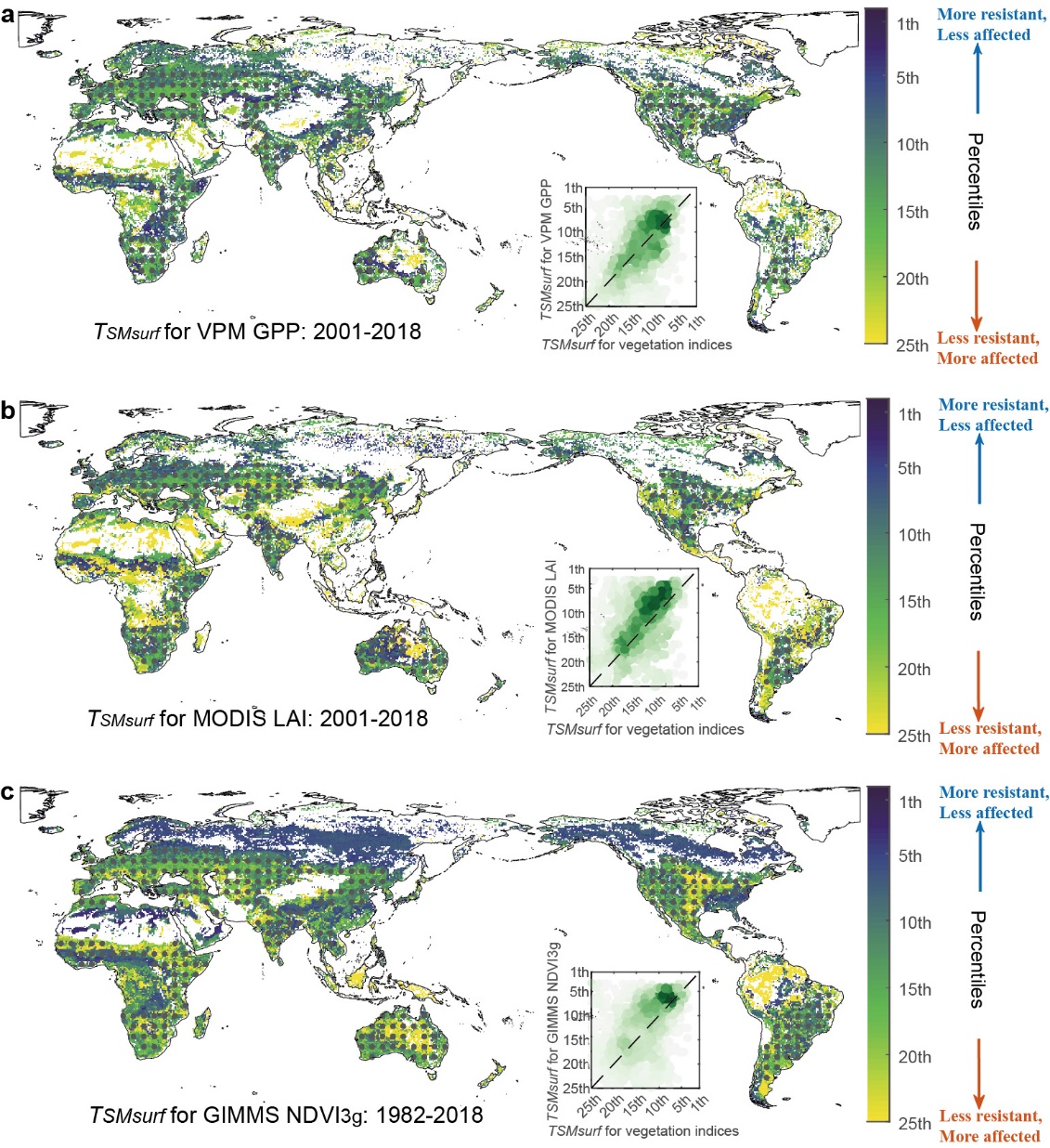


**Supplementary Figure 11. The spatial patterns of drought thresholds for VPM gross primary production (GPP), MODIS LAI and GIMMS NDVI_3g_.** The insert plots show the consistency of *T_SMsurf_* for vegetation indices and VPM GPP (a), MODIS LAI (b), and GIMMS NDVI_3g_ (c). The areas are marked by dots only if the maximum coincidence rate is larger than 0.3 for estimating thresholds.


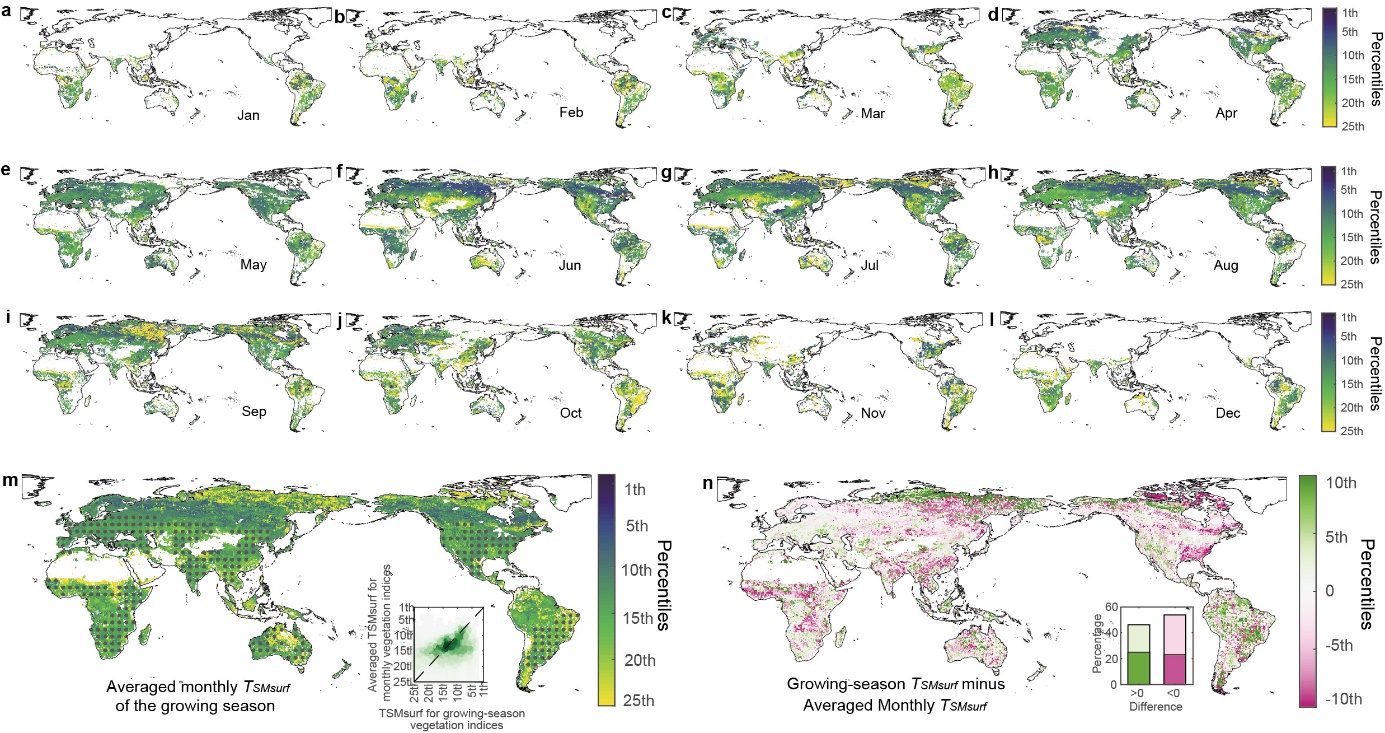


**Supplementary Figure 12.** **Monthly drought thresholds of SMsurf anomalies (*T_SMsurf_*) in the growing season.** **a-l** Monthly *T_SMsurf_* values for individual months of January – December respectively, averaged from threshold values derived with four satellite-based observation datasets (NDVI, kNDVI, NIRv and SIF). The white space means vegetation is not in the growing season so TSMsurf is unable to be identified. **m.** The monthly *T_SMsurf_* values are estimated within each month that is in the growing season for vegetation. The areas averaged by more than one of the satellite data sets or model simulations and the maximum coincidence rates of the samples for estimating thresholds > 0.3 are marked with dots. The inset plot shows the consistency of *T_SMsurf_* derived as a single calculation for the vegetation indices of the growing season, versus the averaged monthly-derived vegetation indices derived from average monthly of the growing season. **n.** The difference between TSMsurf from the growing season and averaged monthly *T_SMsurf_* of the data growing season, and so the map that leads to the inset of panel m. The insert plot of panel n shows the percentage of grid cells with the higher growing-season *T_SMsurf_* than averaged monthly *T_SMsurf_* (>0, green bar chart) and the percentage of grid cells with the lower growing-season *T_SMsurf_* than averaged monthly *T_SMsurf_*, respectively (<0, pink bar chart). The percentages indicated by dark green and dark pink bar charts are calculated using those grid cells marked by dots in **m**.


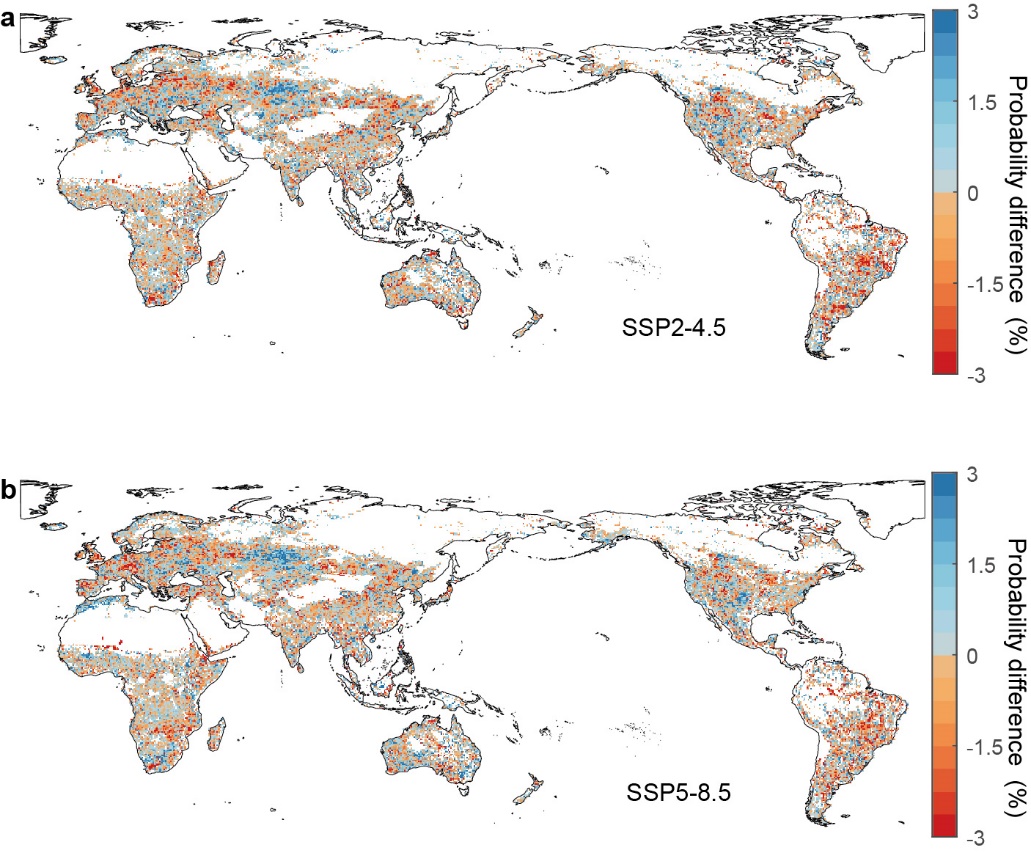


**Supplementary Figure 13.** **Comparison of changes in the probability of occurrence of droughts during 2081-2100 relative to 2001-2020 by using the 10^th^ percentile and the observed-*T_SMsurf_* to define droughts.** The negative and positive values correspond to lower and higher probabilities of drought occurrence identified using the 10^th^ percentile compared to observed-*T_SMsurf_*. Panel (a) is for the SSP2-4.5 future greenhouse gas scenario and panel (b) is for the SSP5-8.5 scenario.


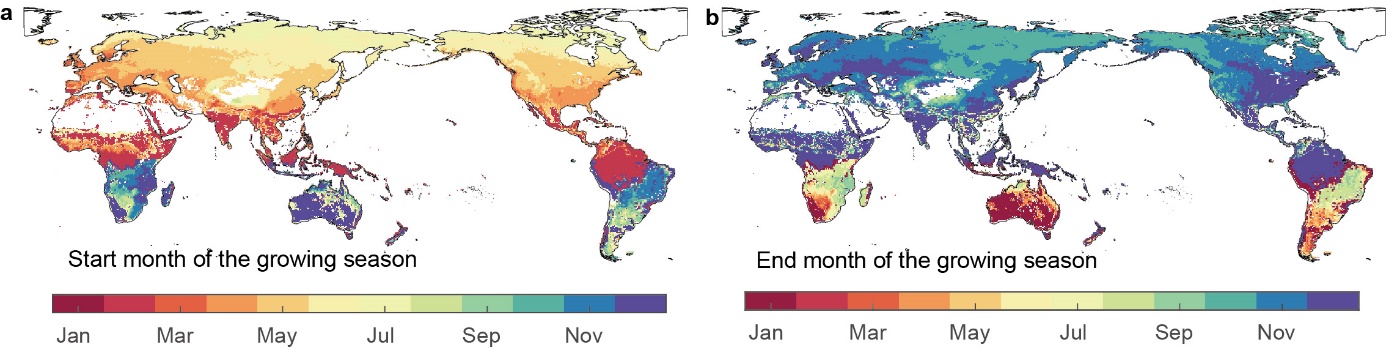


**Supplementary Figure 14. Global maps of the growing season used in this study.** The start and end month of the growing season used in this study. The growing season data set source from Zhu et al., (2013).


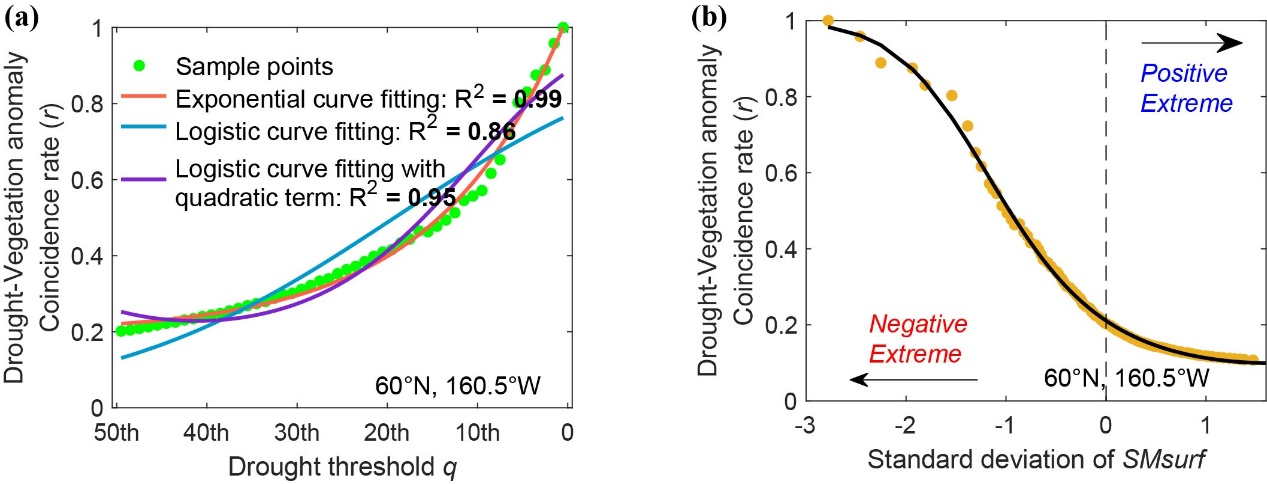


**Supplementary Figure 15. Comparison of different potential response functions.** (a) An example of the response curves fitted by the exponential function (red), the logistic function with the linear term (blue), and the logistic function with the linear and quadratic terms (purple). (b) Fitting of the response curve using soil drought severity as the *x*-axis and the coincidence rate of the drought-vegetation anomaly as the *y*-axis. The grid cell used is located at 60°N, 160.5°W.

**
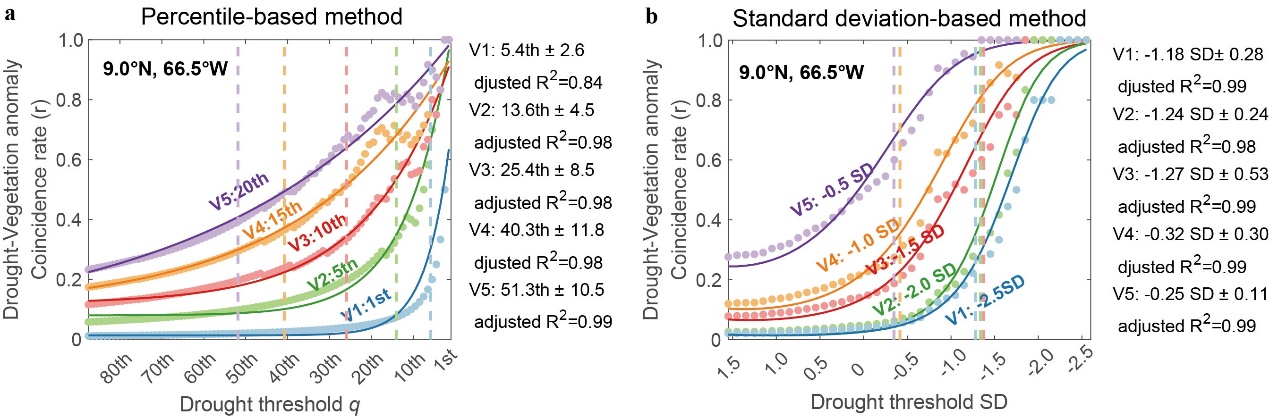
**

**Supplementary Figure 16. An example location (9.0°N, 66.5°W) for observed vegetation NDVI responses to increasing drought thresholds based on the percentile method and standard deviation method.** **a.** Droughts are defined by percentile ranges of the surface soil moisture (SMsurf) anomalies during the growing season while vegetation response is defined by the NDVI anomalous <1^st^ (blue), <5^th^ (green), <10^th^ (red), <15^th^ (orange), and <20^th^ (purple), respectively. The fitted response curves in **a** follow a form of the exponential function, which are fitted by the points sampled at an interval of 1 percentile. The dotted vertical lines display the position of the inflection points detected by all sample points. **b.** Droughts are defined by standard deviation ranges of the SMsurf anomalies during the growing season while vegetation response is defined by the NDVI anomalous < -2.5 SD (blue), <-2.0 SD (green), < -1.5 SD (red), < -1.0 SD (orange), and < -0.5 SD (purple), respectively. The fitted response curves in **b** follow a form of the logistic function, which are fitted by the points sampled at an interval of 0.1SD. The dotted vertical lines display the position of the inflection points detected by all sample points. The texts at the right of the panel displayed the adjusted R square of the expected response curve, the percentiles (a) and standard deviation (b) of the SMsurf anomalies for the inflection points of the samples as well as the uncertainty of percentiles estimated by 1000 times bootstrapping.

**
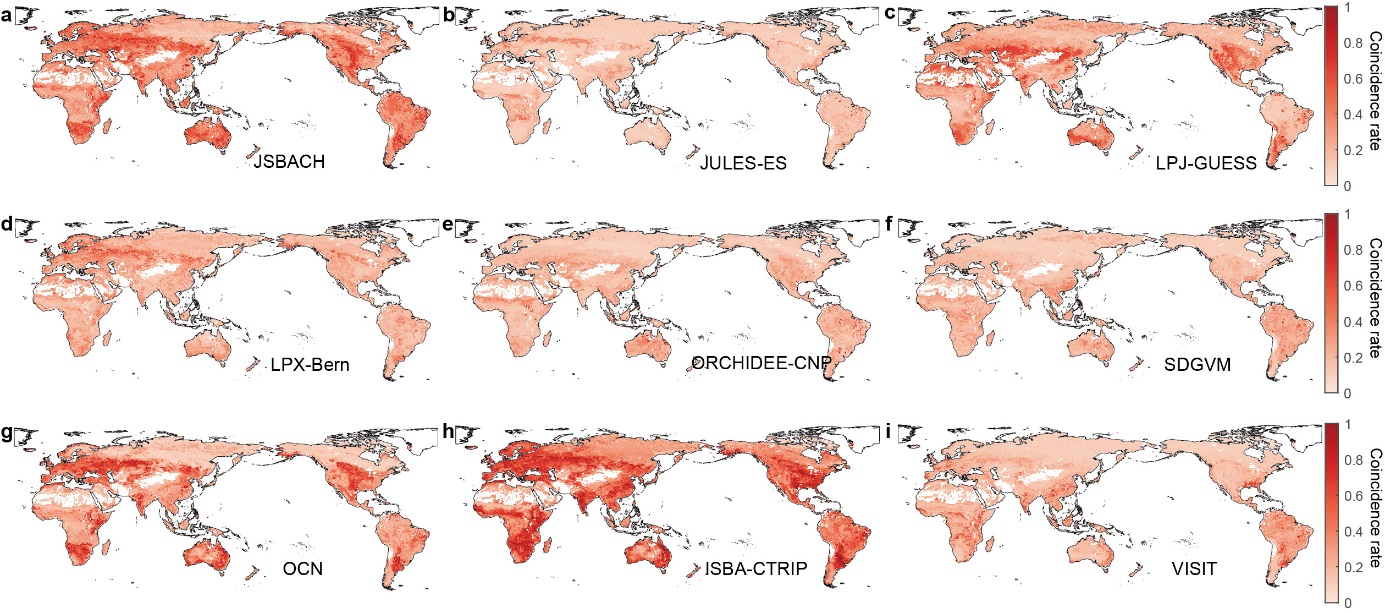
**

**Supplementary Figure 17.** The spatial patterns of maximum coincidence rates using all testing drought stress levels (1^st^ -50^th^ percentile) for model-derived LAI. Nine model simulations are JSBACH, JULES-ES, LPJ-GUESS, LPX-Bern, ORCHIDEE-CNP, SDGVM, OCN, ISBA-CTRIP, and VISIT (a-i). Droughts are identified by anomalies of SMsurf and vegetation response is defined as < 10th percentile of vegetation anomalies.


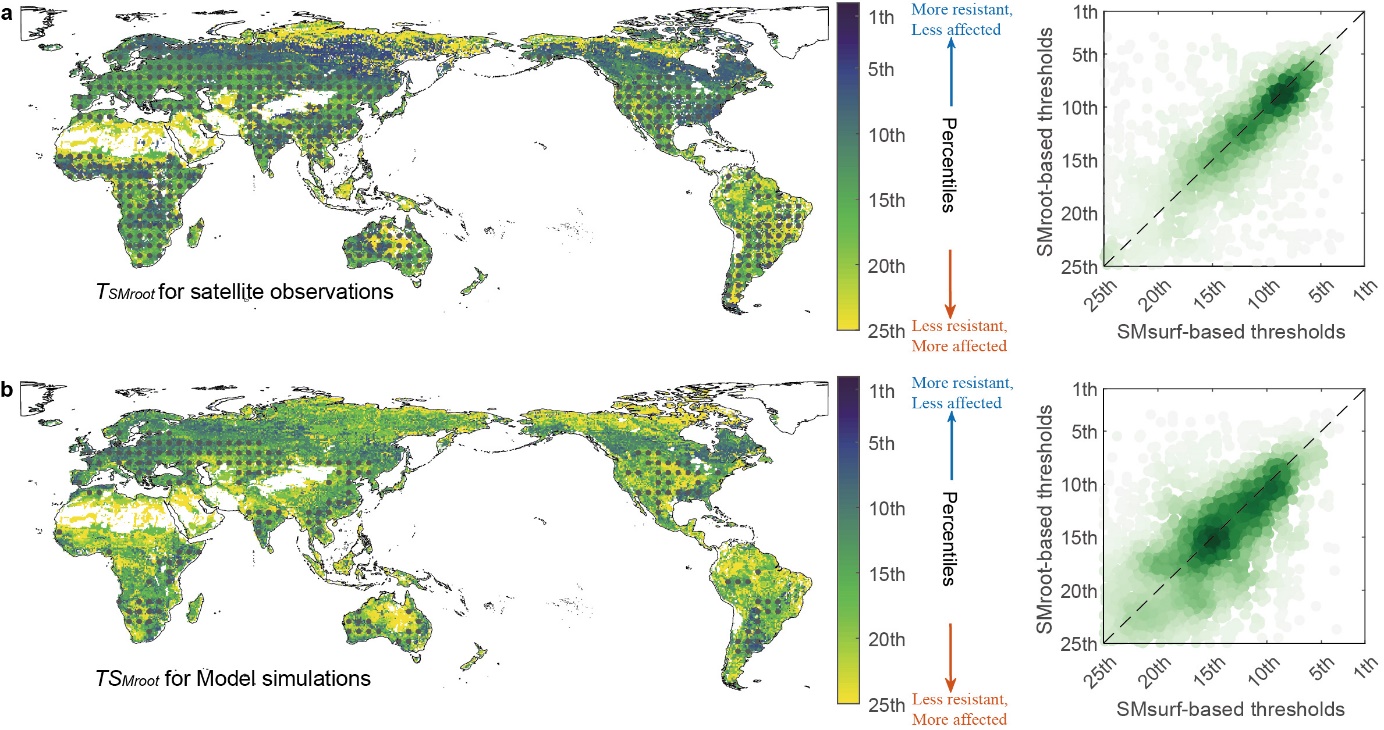


**Supplementary Figure 18.** Spatial patterns of growing-season drought threshold (*T_SMroot_*) of anomalies of SMroot for satellite observations (NDVI, kNDVI, NIRv and SIF) for 2001-2018 (left column). The right column shows the consistency of *T_SMsurf_* and *T_SMroot_* derived using SMsurf (*x*-axis) and SMroot (*y*-axis). Vegetation response is defined as anomalies of the vegetation indicators (NDVI, kNDVI, NIRv and SIF) < 10th percentile.

**Supplementary Tables**

**Supplementary Table 1. List of the nine terrestrial ecosystem models from the TRENDY-v8 data sets.**

| Model | Spatial resolution (latitude × longitude) | | Temporal resolution | Period used in this study | Reference |
| --- | --- | --- | --- | --- | --- |
| JSBACH | 1.875°×1.875° | Monthly | | 2001-2018 | Mauritsen et al. (2018) |
| JULES-ES | 1.25°×1.875° | Monthly | | 2001-2018 | Clark et al. (2011) |
| LPJ-GUESS | 0.5°×0.5° | Monthly | | 2001-2018 | Smith et al. (2014) |
| LPX-Bern | 0.5°×0.5° | Monthly | | 2001-2018 | Lienert and Joos (2018) |
| ORCHIDEE-CNP | 2°×2° | Monthly | | 2001-2018 | Goll et al. (2017) |
| SDGVM | 0.5°×0.5° | Monthly | | 2001-2018 | Walker et al. (2017) |
| OCN | 1°×1° | Monthly | | 2001-2018 | Zaehle and Friend (2010) |
| ISBA-CTRIP | 1.2°×1° | Monthly | | 2001-2018 | Joetzjer et al. (2015) |
| VISIT | 0.5°×0.5° | Monthly | | 2001-2018 | Kato et al. (2013) |

**Supplementary Table 2. List of the Coupled Model Intercomparison Project Phase 6 (CMIP6) models used.**

| Model | Institution ID | Institution |
| --- | --- | --- |
| ACCESS-CM2 | CSIRO-ARCCSS | Commonwealth Scientific and Industrial Research Organisation, Australian Research Council Centre of Excellence for Climate System Science, Australia |
| ACCESS-EMS1-5 | CSIRO | Commonwealth Scientific and Industrial Research Organisation, Aspendale, Australia |
| BCC-CSM2-MR | BCC | Beijing Climate Center, China |
| CESM2-WACCM | NCAR | National Center for Atmospheric Research, USA |
| EC-Earth3 | EC-Earth Consortium | EC-Earth Consortium, EU |
| EC-Earth3-Veg | EC-Earth Consortium | EC-Earth Consortium, EU |
| KACE-1-0-G | NIMS-KMA | National Institute of Meteorological Sciences, Meteorological Administration, Republic of Korea |
| MIROC6 | MIROC | Japan Agency for Marine-Earth Science and Technology, Atmosphere and Ocean Research Institute, National Institute for Environmental Studies, RIKEN Center for Computational Science, Japan |
| MPI-ESM1-2-LR | MPI | Max Planck Institute for Meteorology, Germany |
| MPI-ESM1-2-HR | MPI | Max Planck Institute for Meteorology, Germany |
| MRI-ESM2-0 | MRI | Meteorological Research Institute, Japan |
| NorESM2-MM | NCC | Center for International Climate and Environmental Research, Norwegian Meteorological Institute, Nansen Environmental and Remote Sensing Center, Norwegian Institute for Air Research, University of Bergen, University of Oslo, Uni Research, Norway |

**Supplementary Table 3 The information of seven variables used in the principal component analysis.**

| No | Abbreviation | Variable | Variable type | Source |
| --- | --- | --- | --- | --- |
| 1 | MAP | Mean annual precipitation | Climate | CRU TS v4, Harris et al., 2020 |
| 2 | MAT | Mean annual temperature | Climate |  |
| 3 | VPDvar | Annual variability of vapor pressure deficit | Climate |  |
| 4 | NDVIvar | Annual variability of NDVI | Vegetation | MODIS |
| 5 | NIRvvar | Annual variability of NIRv | Vegetation | MODIS |
| 6 | Treefrac | Fraction of tree cover | Vegetation | Li et al. 2016 |
| 7 | SpeciesN | Species richness | Ecosystem composition | Ellis et al., 2012 |

**Reference**

Cook, B. I. , Mankin J S, Marvel K *et al.* Twenty‐First Century Drought Projections in the CMIP6 Forcing Scenarios. *Earth’s Future* 2020; 8(6): 2328-4277.

Anderegg, W. R. L. , Schwalm C, Biondi F *et al.* Pervasive drought legacies in forest ecosystems and their implications for carbon cycle models. Science 2015; 349(6247): 528–532.

Huang, M., Wang, X., Keenan, T. F. *et al*. Drought timing influences the legacy of tree growth recovery. *Glob Change Biol* 2018; 24: 3546–3559.

Mauritsen, T., Bader, J., Becker, T. *et al*. Developments in the MPI‐M Earth System Model version 1.2 (MPI‐ESM 1.2) and its response to increasing CO_2_. *J. Adv. Model. Earth Sy.* 2018; 11, 998– 1038.

Clark, D. B., Mercado, L. M., Sitch, S. *et al*. The Joint UK Land Environment Simulator (JULES), model description–Part 2: carbon fluxes and vegetation dynamics. *Geoscientific Model* Development, 2011; **4**: 701-722.

Smith, B., Warlind, D., Arneth, A. *et al*. Implications of incorporating N cycling and N limitations on primary production in an individual-based dynamic vegetation model. *Biogeosciences*, 2014; 11: 2027-2054.

Lienert, S., & Joos, F. A Bayesian ensemble data assimilation to constrain model parameters and land-use carbon emissions. *Biogeosciences*, 2018; *15*: 2909-2930.

Goll, D., Vuichard, N., Maignan, F. *et al*. A representation of the phosphorus cycle for ORCHIDEE (revision 4520). *Geoscientific Model Development Discussions* 2017; 10:3745-3770.

Walker, A. P., Quaife, T., van Bodegom, P. M. *et al*. The impact of alternative trait‐scaling hypotheses for the maximum photosynthetic carboxylation rate (Vcmax) on global gross primary production. *New Phytol.* 2017; 215: 1370-1386.

Zaehle, S. and Friend, A. D. Carbon and nitrogen cycle dynamics in the O-CN land surface model: 1. Model description, site-scale evaluation, and sensitivity to parameter estimates, *Global Biogeochem. Cy.*, 2010; 24: GB1005.

Joetzjer, E., Delire, C., Douville, H. *et al*. Improving the ISBA (CC) land surface model simulation of water and carbon fluxes and stocks over the Amazon forest. *Geoscientific Model Development*, 2015: 8: 1709-1727.

Kato, E., Kinoshita, T., Ito, A., *et al*. Evaluation of spatially explicit emission scenario of land-use change and biomass burning using a process-based biogeochemical model. *J. Land Use Sci.* 2013; 8: 104-122.

Harris, I., Osborn, T. J., Jones, P. *et al*. Version 4 of the CRU TS monthly high-resolution gridded multivariate climate dataset. Sci Data 2020; 7: 109.

Li, W., Ciais, P., MacBean, N. *et al*. Major forest changes and land cover transitions based on plant functional types derived from the ESA CCI Land Cover product, *Int. J. Appl. Earth Obs.*2016; 47: 30–39.

Ellis, E. C., Antill, E. C. & Kreft, H. All Is Not Loss: Plant Biodiversity in the Anthropocene. *PLoS ONE* 2012; 7: e30535.
